# Understanding zwitterionic ring‐expansion polymerization through mass spectrometry

**DOI:** 10.1002/mas.21877

**Published:** 2024-03-31

**Authors:** Mahi Ahmad, Scott M. Grayson

**Affiliations:** ^1^ Department of Chemistry Tulane University New Orleans Louisiana USA

**Keywords:** mass spectrometry, matrix‐assisted laser desorption/ionization time‐of‐flight mass spectrometry, mechanism, ring‐opening polymerization, zwitterionic ring‐expansion polymerization

## Abstract

Zwitterionic ring‐expansion polymerization (ZREP) is a polymerization method in which a cyclic monomer is converted into a cyclic polymer through a zwitterionic intermediate. In this review, we explored the ZREP of various cyclic polymers and how mass spectrometry assists in identifying the product architectures and understanding their intricate reaction mechanism. For the majority of polymers (from a few thousand to a few million Da) matrix‐assisted laser desorption/ionization time‐of‐flight mass spectrometry is the most effective mass spectrometry technique to determine the true molecular weight (MW) of the resultant product, but only when the dispersity is low (approximately below 1.2). The key topics covered in this study were the ZREP of cyclic polyesters, cyclic polyamides, and cyclic ethers. In addition, this study also addresses a number of other preliminary topics, including the ZREP of cyclic polycarbonates, cyclic polysiloxanes, and cyclic poly(alkylene phosphates). The purity and efficiency of those syntheses largely depend on the catalyst. Among several catalysts, *N*‐heterocyclic carbenes have exhibited high efficiency in the synthesis of cyclic polyesters and polyamides, whereas tris(pentafluorophenyl)borane [B(C_6_F_5_)_3_] is the most optimal catalyst for cyclic polyether synthesis.

**Abbreviations**
APCI‐MS: atmospheric pressure chemical ionization mass spectrometry
Ð: dispersity
DP: degree of polymerization
ESI‐MS: electrospray ionization mass spectrometry
IMS‐MS: ion mobility spectrometry mass spectrometry
MALDI‐TOF MS/MALDI‐MS: matrix‐assisted laser desorption/ionization time‐of‐flight mass spectrometry
MW: molecular weight
NMR: nuclear magnetic resonance
REP: ring‐expansion polymerization
ROP: ring‐opening polymerization
SEC: size exclusion chromatography
SGCC: silica gel column chromatography
ZREP: zwitterionic ring‐expansion polymerization

## 1 INTRODUCTION

**Scheme 1:**
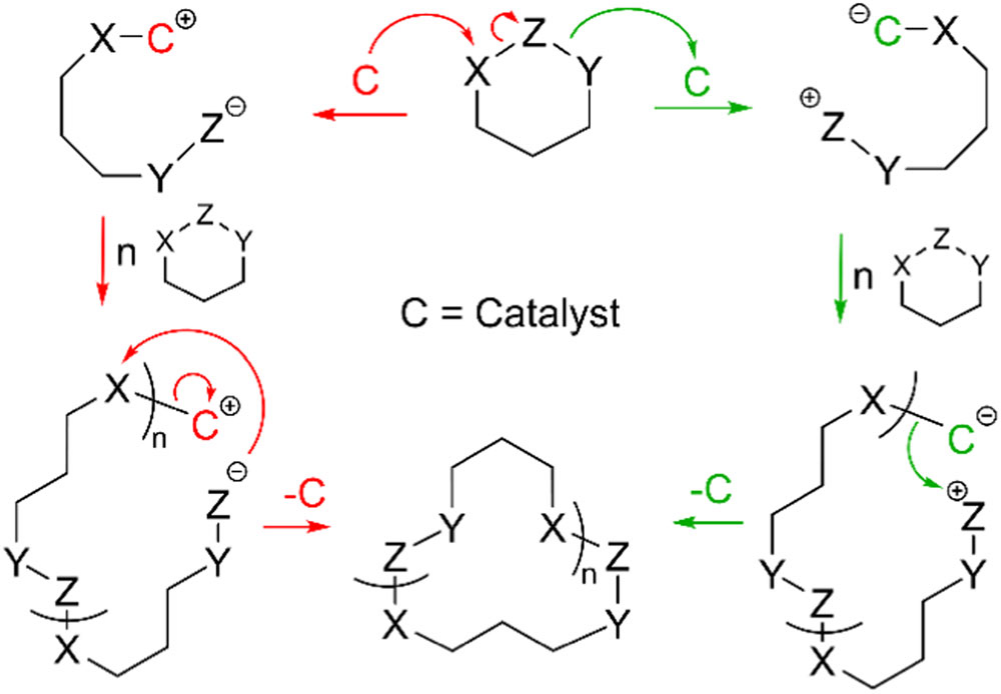
Zwitterionic ring‐expansion polymerization mechanism of cyclic polymer. [Color figure can be viewed at wileyonlinelibrary.com]

Ring‐opening polymerization (ROP) is a chemical reaction in which an initiator cleaves the single weakest bond of a cyclic monomer to initiate polymerization. Following the initiation step, one of the end groups of the linear polymer can attack numerous other cyclic monomers, which will propagate until the reaction terminates to form a larger linear polymer (Albertsson & Varma, 2003; Kamber et al., 2007). For instance, many of these monomers can react with adventitious water, which can act as an initiator, and the resulting polymer will have a hydroxyl group on the initiating end and a hydrogen group appended to the weakest bond on the other end. However, without a trace of water or another initiator, some catalysts can expand the cyclic monomer to produce larger cyclic polymers. These polymerizations are referred to as ring‐expansion polymerization (REP) (Chang & Waymouth, 2017; Xia et al., 2009). There are different types of REP, such as zwitterionic ring‐expansion polymerization (ZREP), radical ring‐expansion polymerization, ring‐expansion metathesis polymerization, and so forth (Chang & Waymouth, 2017). This review only focuses on the ZREP and how mass spectrometric analysis can determine this polymerization's mechanism. In ZREP, a cyclic monomer will react with a catalyst to form a zwitterionic intermediate. The presence of a negative anion on one side and a positive cation on the other side leads to the formation of an ionic bond. Subsequently, depending on the nature of the monomer, either the cation or anion of the zwitterionic compound will attack another cyclic monomer and continue to propagate forming a larger cyclic intermediate. However, eventually at some point during the reaction, the negative site of this zwitterion will attack the positive site adjacent to the catalyst (or vice versa). As a result of the attack, the catalyst will be eliminated by forming a covalent bond to yield a cyclic polymer (Scheme 1).

The characterization of cyclic polymer exhibits many challenges, including differentiating between cyclic and linear counterparts, determining the completion of cyclization, identifying product degradation, and so forth (Chan et al., 2009; Haque & Grayson, 2020; Hoskins & Grayson, 2009; Hoskins et al., 2011; Nasongkla et al., 2009). Several characterization techniques have been combined to determine the architecture, such as nuclear magnetic resonance (NMR), tandem mass spectrometry (MS/MS), matrix‐assisted laser desorption/ionization time‐of‐flight mass spectrometry (MALDI‐TOF MS), electrospray ionization mass spectrometry (ESI‐MS), size‐exclusion chromatography (SEC), silica gel column chromatography (SGCC), and so forth (Grayson & Fréchet, 2002; Haque & Grayson, 2020; Hoskins & Grayson, 2009; Nitsche et al., 2021). For the rest of this article, MALDI‐TOF MS will be referred to as MALDI‐MS. Among these characterization techniques, mass spectrometry is probably the most practical for determining this particular cyclic polymer topology. However, not all of the MS techniques are suitable for determining various information regarding polymer synthesis after a certain MW. For example, electron impact, chemical ionization, or fast atom bombardment mass spectrometry can only volatilize low MW oligomers. In case of ESI‐MS, it is possible to detect higher MW, but the sample above *m/z* = 2500 Da may ionize with multiple charges. For instance, if the average MW of a compound is 3600 Da, it may show peaks at *m/z* (mass‐to‐charge ratio) = 1800, 1200, 900, and so forth for *z* (charge on ions) = 2, 3, 4, and so forth. Due to the larger polymer's ability to ionize with different charge states, the spectrum may have multiple distributions rather than a single distribution. However, with modern ESI, it is possible to deconvolute these data and determine the true molecular weight of the polymer. Conversely, on MALDI‐MS, samples ionize predominantly with a single charge, yet occasionally they may give a double charge with a higher MW (Seyfried et al., 2010; Xuan et al., 2000). MALDI‐MS can detect polymers with a much higher MWs (as high as a few million Da) (Schriemer & Li, 1996); however, there can be complications below 1000 Da from the matrix‐related signals. Although MALDI‐MS is a highly effective technique, it cannot differentiate between isomeric linear and cyclic polymeric architecture (Baytekin et al., 2006; Felder et al., 2005; Yol & Wesdemiotis, 2014). To overcome this challenge, tandem mass spectrometry (MS/MS) can be utilized to identify the different polymeric architectures (Arnould et al., 2005; Wesdemiotis et al., 2006; Wollyung et al., 2004, 2005). One of the most common MS/MS investigations involves the selection of a specific precursor ion from those generated through MALDI or ESI techniques, followed by fragmentating the selected ion. To deduce the structure of selected ion from the fragment masses, it is essential to understand the fragmentation patterns that led to their dissociation. By utilizing the established fragmentation routes, it is possible to accurately reconstruct the fragments and deduce the corresponding structural configuration, including linear, cyclic, and branched polymers (Mao et al., 2019; Wesdemiotis et al., 2011; Yol & Wesdemiotis, 2014). However, when the structural details cannot be definitely determined through the fragmentation resulting from MS/MS, ion mobility spectrometry‐mass spectrometry (IMS‐MS) can serve as a valuable alternative. The IMS‐MS technique facilitates the gas‐phase separation of ions by considering their charge states and cross‐sectional properties which are basically the size and shape of the polymer. IMS‐MS data allows for the direct association between the mass‐to‐charge ratio (*m/z*) and the size of each macromolecule, as indicated by its drift time (Chan et al., 2009; Clemmer & Jarrold, 1997; Hoskins et al., 2011). For instance, the drift time of the cyclic polymer will be shorter than that of the linear counterpart for identical charge states, due to its more condensed structure (Hoskins et al., 2011; Wilkins & Trimpin, 2011).

The following sections will briefly discuss the contributions and limitations of NMR, SGCC, and SEC data and how MALDI‐MS can overcome most of the limitations for characterizing cyclic polymers.

NMR spectra may help us distinguish between cyclic and linear analogs for low MW polymers since cyclic polymers only show repeating unit peaks, whereas linear polymers exhibit the end group peaks as well. However, this differentiation between polymer architectures decreases as we get to higher molecular weight (>25 kDa) (Izunobi & Higginbotham, 2011). This is due to the fact that in linear polymers, the peaks corresponding to the end groups become indistinguishable from background noise at high MWs, posing a challenge in accurately determining the true architecture of the polymer. Moreover, NMR can only estimate the MWs of pure linear polymers, not cyclic polymers or mixtures of cyclic and linear polymers. For instance, if a polymer chemist synthesizes a pure cyclic polymer, the NMR spectrum cannot determine the MW of the product since there are no end groups peaks to compare with repeating unit peaks. Now, if the chemist has a mixture of cyclic and linear products, the NMR spectrum will display peaks for both repeating unit (cyclic and linear) and end group (linear), making it impossible to infer the ratio of cyclic and linear for those peaks. Furthermore, sometimes the peaks of the repeating unit and the end groups overlap, which might lead to a false identification of a cyclic product. On the other hand, MALDI‐MS in combination with MS/MS fragmentation has the ability to precisely define the end groups of the polymers (or absence thereof), which aids in differentiating the pure and impure products. Mass spectrometric data can also tell us about different fractions of linear and cyclic for each MW (if they have a change in their mass distribution). Another disadvantage of NMR is that it cannot readily identify the architectural impurities including tadpoles, branches, and so forth whereas MALDI‐MS can detect the exact MW (especially when the polydispersity is less than 1.2) of multiple impurities simultaneously in one spectrum. However, if either the linear polymer or the cyclic polymer has a greater ionization efficiency than the other, this can skew the data.

It is complicated to identify the impurities only through SGCC technique. SGCC can separate and identify different polymer architectures and impurities with the help of other techniques like NMR, mass spectrometry, SEC, and so forth. In other words, after SGCC separation of different fractions based on their affinity towards the mobile and stationary phase, mass spectrometric data can reveal insight about the MWs of those fractions, which can be used to speculate about their architecture (Leon et al., 1996; Zhang et al., 2001). Whereas the NMR spectrum can eliminate some of the predicted structures from mass spectrometric analysis, SEC will shed more light on the architecture based on the hydrodynamic volume of the molecule, such as a linear compound and a cyclic compound.

SEC can confirm the completion of the cyclization based on a reduction in hydrodynamic volume (Alberty et al., 2005). However, SEC denotes a change in hydrodynamic volume, which may also signify smaller sizes including branching (Hernández et al., 2008). MALDI‐MS, on the other hand, can verify the polymer's MW and probe the architecture with the help of SEC. Additionally, MALDI‐MS can provide insight about the polymer degradation mechanism by measuring the MW, whereas SEC primarily indicates a rise in dispersity (Ð) and a decrease in M_*n*_.

While many recent studies use ^1^H and ^13^C NMR, SEC, or SGCC, if not all three, MALDI‐MS emerges as the most powerful and effective tool for investigating and comprehending the intricate mechanistic processes involved in polymer synthesis due to its ability to provide critical information regarding end‐groups, structure, and MW for all distributions present within a complex polymeric mixture (Cortez & Grayson, 2010a; Cortez et al., 2015; Hart‐Smith et al., 2009; Liu et al., 2019; Stukenbroeker et al., 2015; Williams et al., 1997). Furthermore, by performing MALDI‐MS analysis on the aliquot during the experiment, it is possible to identify the intermediates and monitor the progression of the reaction (Cortez & Grayson, 2010b; Kelly et al., 2017). In recent years, several articles were published regarding ZREP where researchers used MS as a main tool for the identification of the polymer as well as for predicting a probable mechanism (Chang & Waymouth, 2015; Guo & Zhang, 2009; Kricheldorf & Petermann, 2001). In this review, the mechanistic pathways will be explored and the purity of the products will be studied for the different ZREPs, under the influence of different catalysts, primarily based on MALDI‐MS analysis.

## 2 ZREP OF CYCLIC POLYESTERS

**Figure 1:**
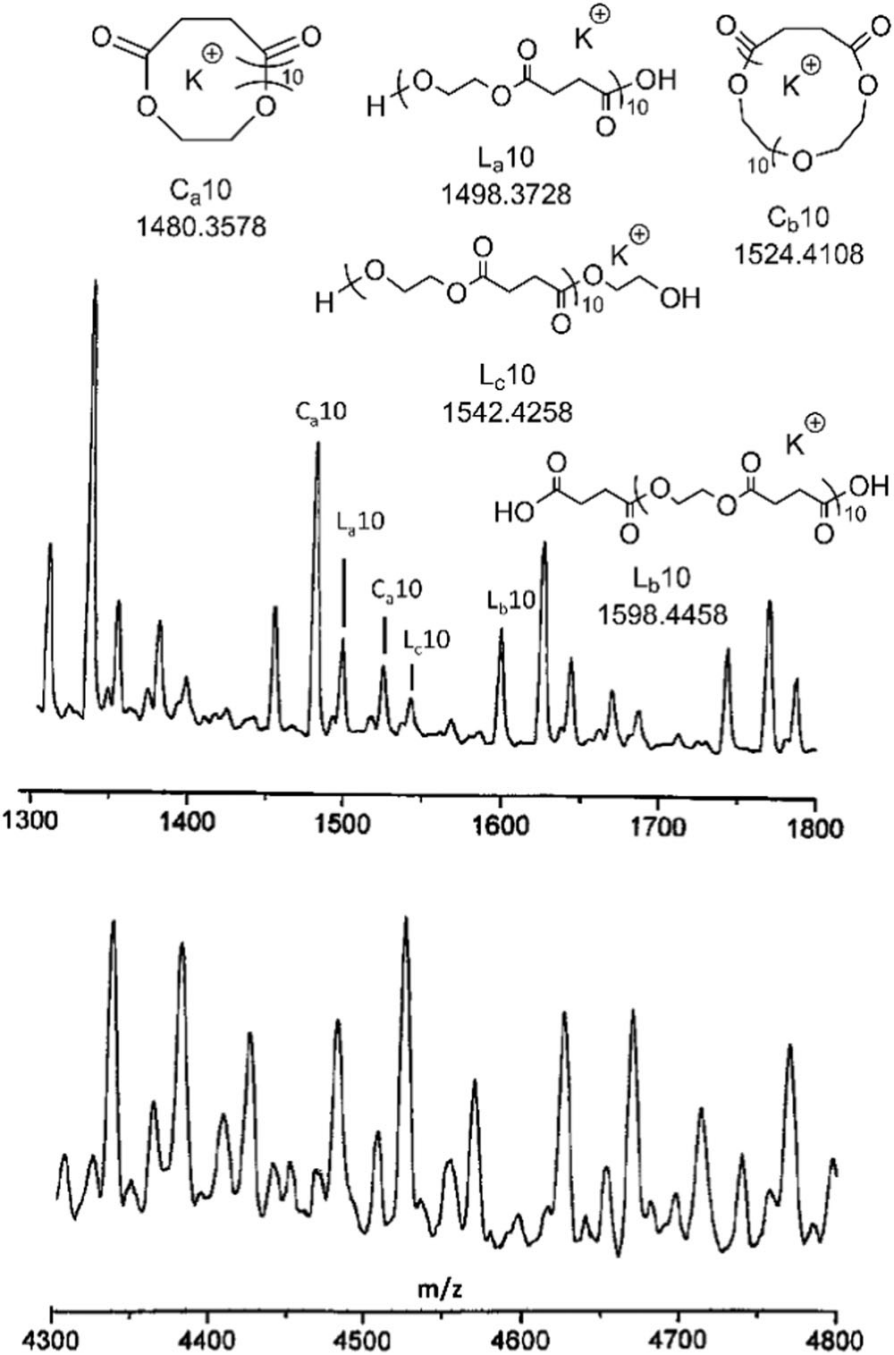
Matrix‐assisted laser desorption/ionization‐mass spectrometry spectrum of poly(ethylene succinate) in presence quinoline. Adapted from Kricheldorf and Petermann (2001) with ACS's permission.

**Scheme 2:**
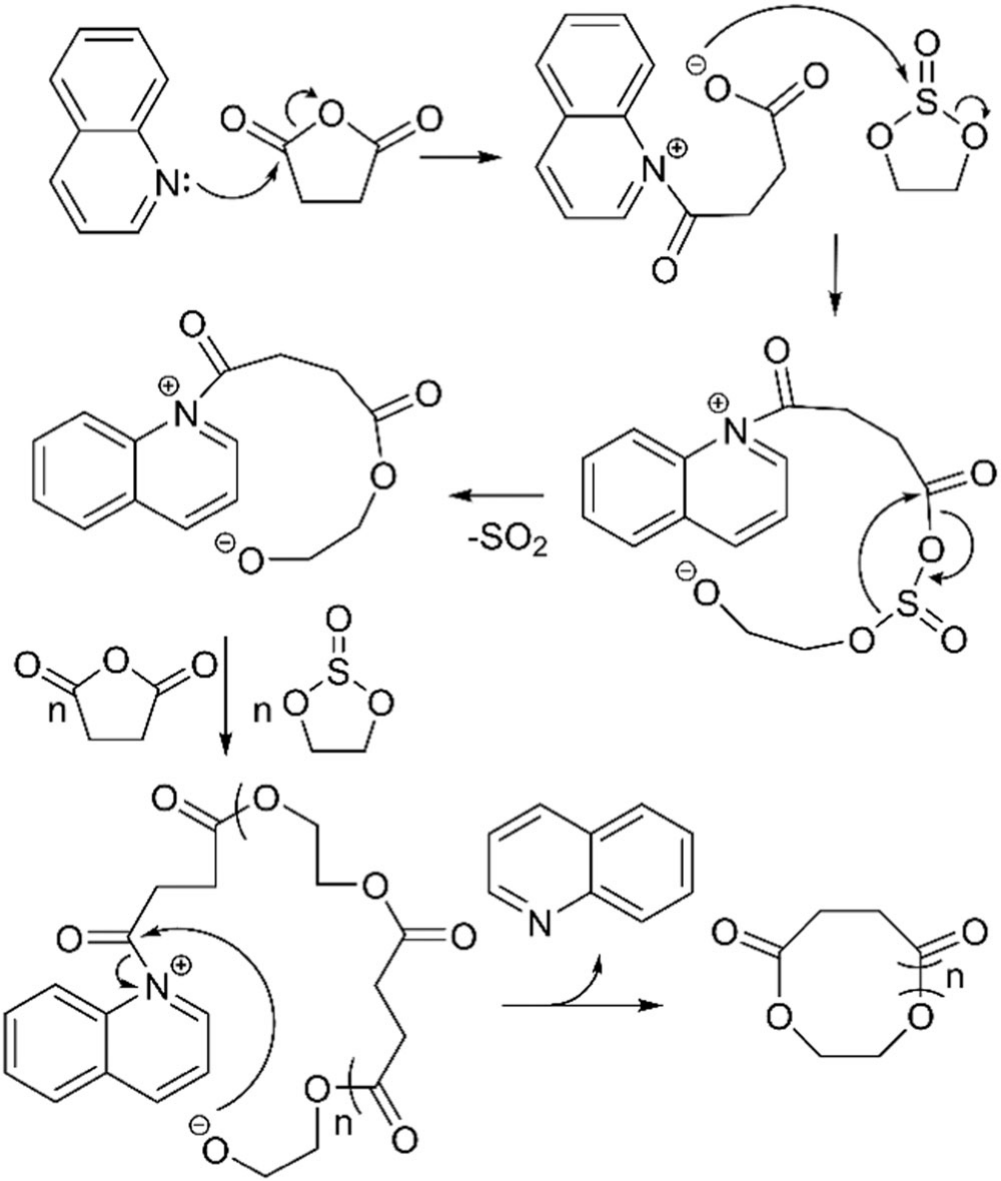
Sulfinylation‐based mechanism while using quinoline as catalyst. Adapted from Kricheldorf and Petermann (2001) with ACS's permission.

**Scheme 3:**
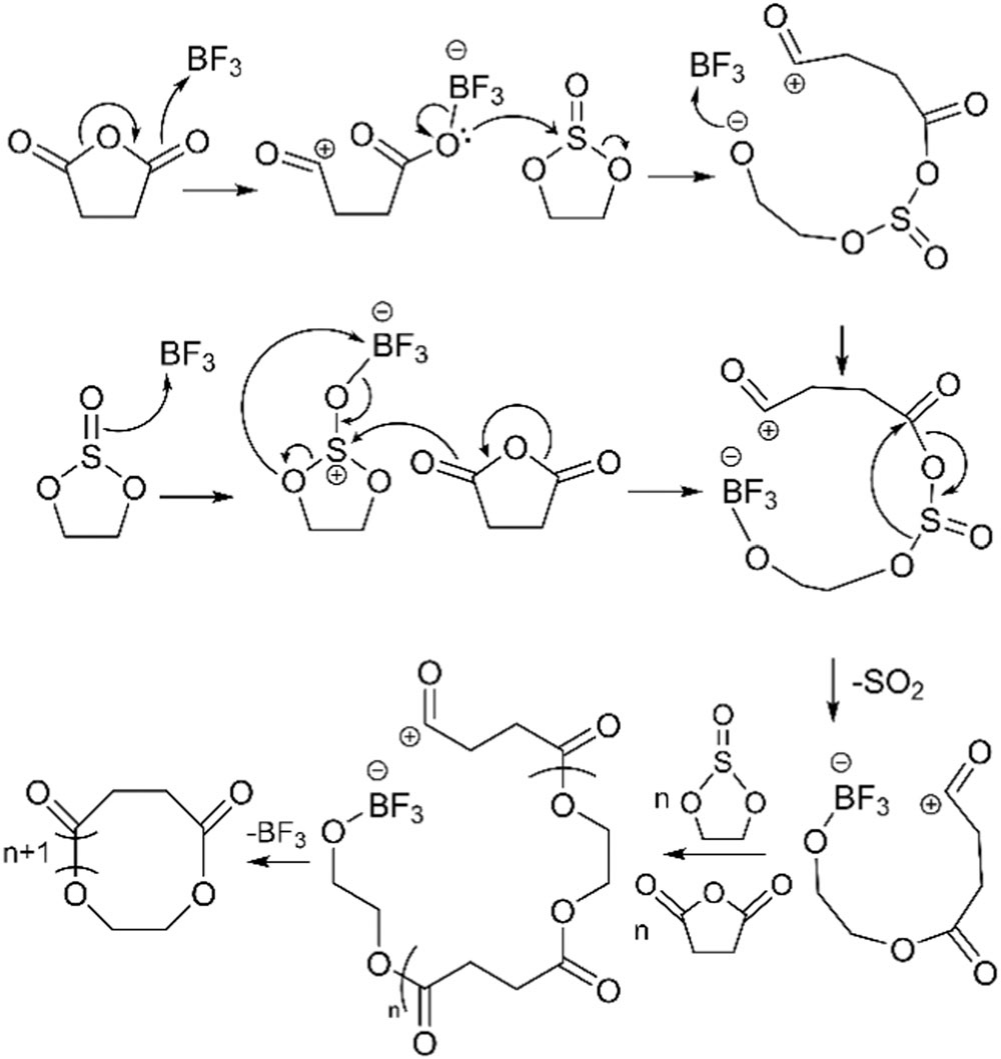
Sulfinylation‐based mechanism while using BF_3_:OEt_2_ as catalyst. Adapted from Kricheldorf and Petermann (2001) with ACS's permission.

In 2001, the very first ZREP of cyclic polyester was reported by Kricheldorf and Petermann (Kricheldorf & Petermann, 2001). Although most of the recent studies have used lactone and lactide as monomers for the cyclic polyester synthesis via‐ZREP, Kricheldorf et al. described cyclic polyesters from cyclic anhydrides and ethylene sulfite. They investigated the polycondensation reactions between ethylene sulfite and six different cyclic anhydrides (succinic anhydride, glutaric anhydride, tetramethylene glutaric anhydride, adipic anhydride, sebacic anhydride, and thiosuccinic anhydride) while using either quinoline or boron trifluoride etherate (BF_3_:OEt_2_) as the catalyst at 180°C for 16 h. To detect the molecular structure of the products, they characterized the resulting polyesters by MALDI‐MS. A mixture of two cyclic polyesters (C_a_ and C_b_), three linear polyesters (L_a_, L_b_, and L_c_), and some unknown products were found in all reactions (Figure 1). In BF_3_:OEt_2_ catalyzed reaction, lower MW cyclic polyesters were the primary product below 2000 Da, but above 4000 Da, the linear and cyclic polymer ratios were approximately the same (based on the intensity of peaks on MALDI‐MS). In case of quinoline‐catalyzed reaction, products had both linear and cyclic mixtures as well (Figure 1). Two primary mechanisms were discussed: sulfinylation of the cyclic anhydrides followed by removal of SO_2_ to form the ester (Schemes 2 and 3) or simultaneous alkylation of the cyclic anhydrides and ester formation. Since the absence of thioester groups in polycondensation products reduces the possibility for propagation via “alkylation” (later of the two‐mechanism proposed above), the sulfinylation‐based mechanism (Schemes 2 and 3) is preferred. Additionally, individual reactions of each monomer with each catalyst were studied to examine the initial step of the polymerization. Upon analyzing the ^1^H and ^13^C NMR data, it was evident that heterocyclic cleavage of cyclic anhydride initiated the reaction when quinoline was used as the catalyst (Scheme 2). Conversely, in the case of boron trifluoride etherate, no considerable evidence was found for the initial step, which implies the possibility of heterocyclic cleavage for both cyclic anhydride and ethylene sulfite as the start point of the reaction (Scheme 3). Moreover, to determine whether the mechanism of the cyclic product was transesterification/backbiting degradation or cyclization of zwitterionic chains, an alternating copolyester (Shaik et al., 2001) was analyzed under the same reaction conditions. ^1^H and ^13^C NMR revealed that the quinoline product only has minor transesterification, though the BF_3_ etherate product shows randomization of the copolyester, implying that the majority of cyclic polymers can be formed through backbiting.

### 2.1 ZREP of lactones

**Scheme 4:**
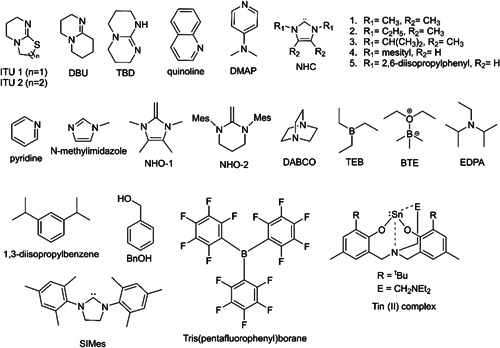
Catalyst discussed in this review.

Waymouth and coworkers have contributed substantially to the field of ZREP of cyclic polyesters from lactones. They have done several studies (Chang & Waymouth, 2015; Jeong et al., 2007; Shin, Brown, et al., 2011; Shin, Jeong, et al., 2011) in this field using four lactones [β‐propiolactone, β‐butyrolactone, ε‐caprolactone (ε‐CL), and δ‐valerolactone (δ‐VL)] as monomers. For achieving polyesters from lactones, different *N*‐heterocyclic carbenes (NHC) were used as the catalyst (Scheme 4). All those studies will be discussed below.

**Scheme 5:**
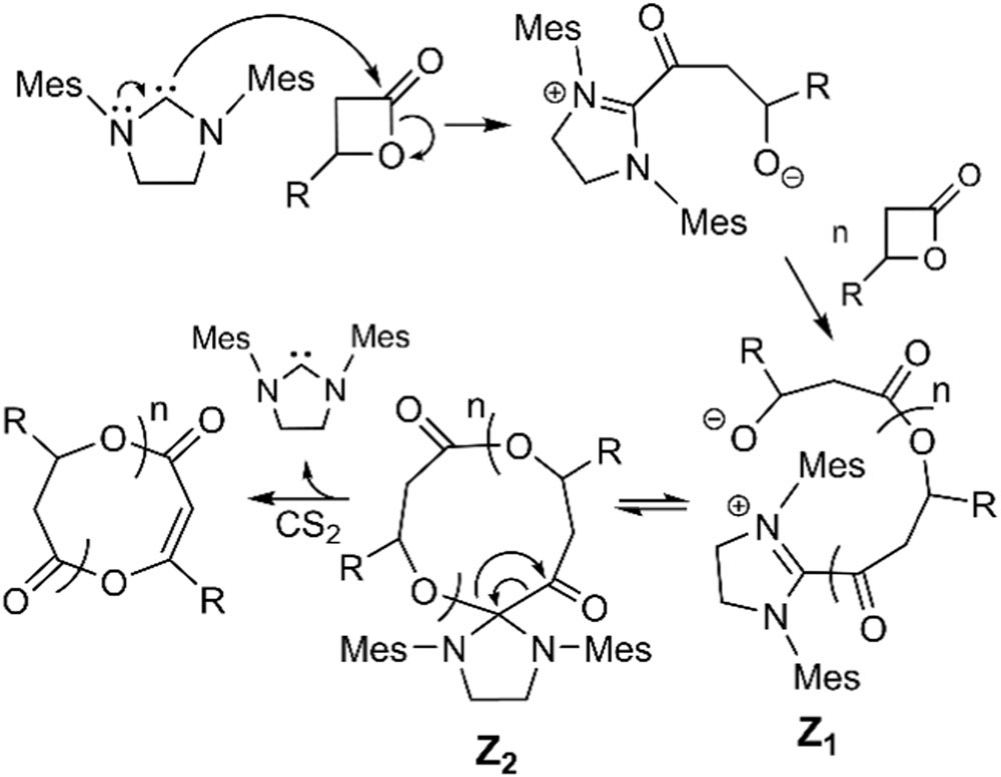
Mechanism of 1,3‐dimesitylimidazol‐2‐ylidene mediated zwitterionic ring‐expansion polymerization. Adapted from Jeong et al. (2007) with ACS's permission.

**Figure 2:**
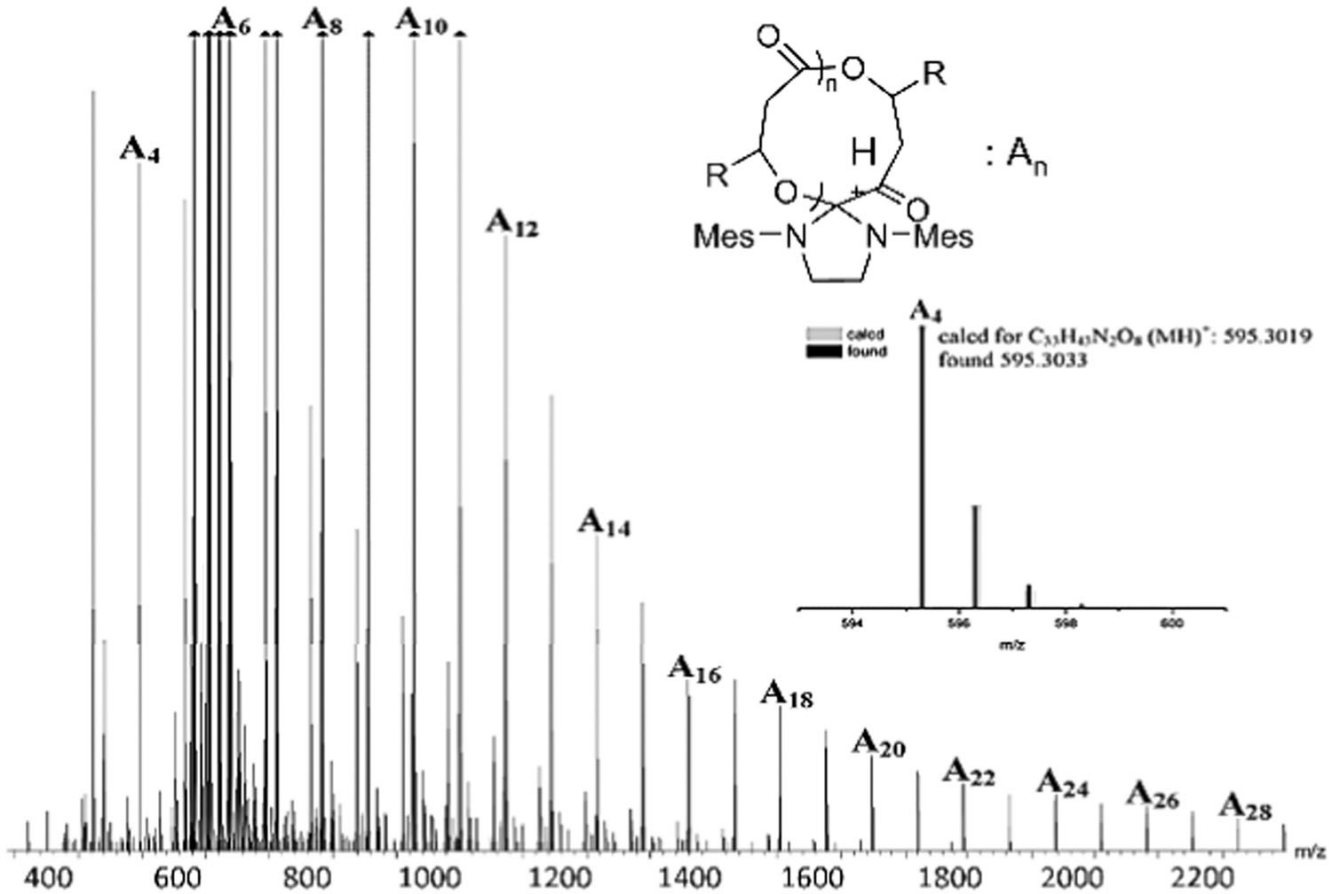
Electrospray ionization mass spectrometry of macrocyclic spirocycles. Adapted from Jeong et al. (2007) with ACS's permission.

Polymerization of β‐propiolactone and β‐butyrolactone were done in the presence of carbene 1,3‐dimesitylimidazol‐2‐ylidene (SIMes) (Scheme 4) as the catalyst to generate cyclic polyester by Jeong et al. (2007) In this work, they claim that the mechanism involves reversible conversion (Scheme 5) of zwitterionic intermediates **Z**_**1**_ into imidazolidine spirocycles **Z**_**2**_ and the cyclic polyester was obtained only after addition of CS_2_ which eliminated the SIMes. The presence of imidazolidine spirocycles **Z**_**2**_ was confirmed by the ESI‐MS spectrum of an aliquot collected during the reaction, where the majority of peaks corresponded to macrocyclic spirocycles (Figure 2). This provided evidence for the equilibrium between the zwitterionic propagation species **Z**_**1**_ and the spirocycles **Z**_**2**_. Moreover, ^1^H NMR and ESI‐MS spectra show the retention of cyclic poly[(R)‐β‐butyrolactone] configuration, which suggests the cyclic product was formed through acyl‐oxygen cleavage. Based upon the ESI‐MS data, the β‐propiolactone generates predominantly cyclic polyester, whereas β‐butyrolactone produces a mixture of cyclic and other unknown products. In both cases, the impure cyclic product was formed between MW 1000–4000 Da with dispersity below 1.3.

**Scheme 6:**
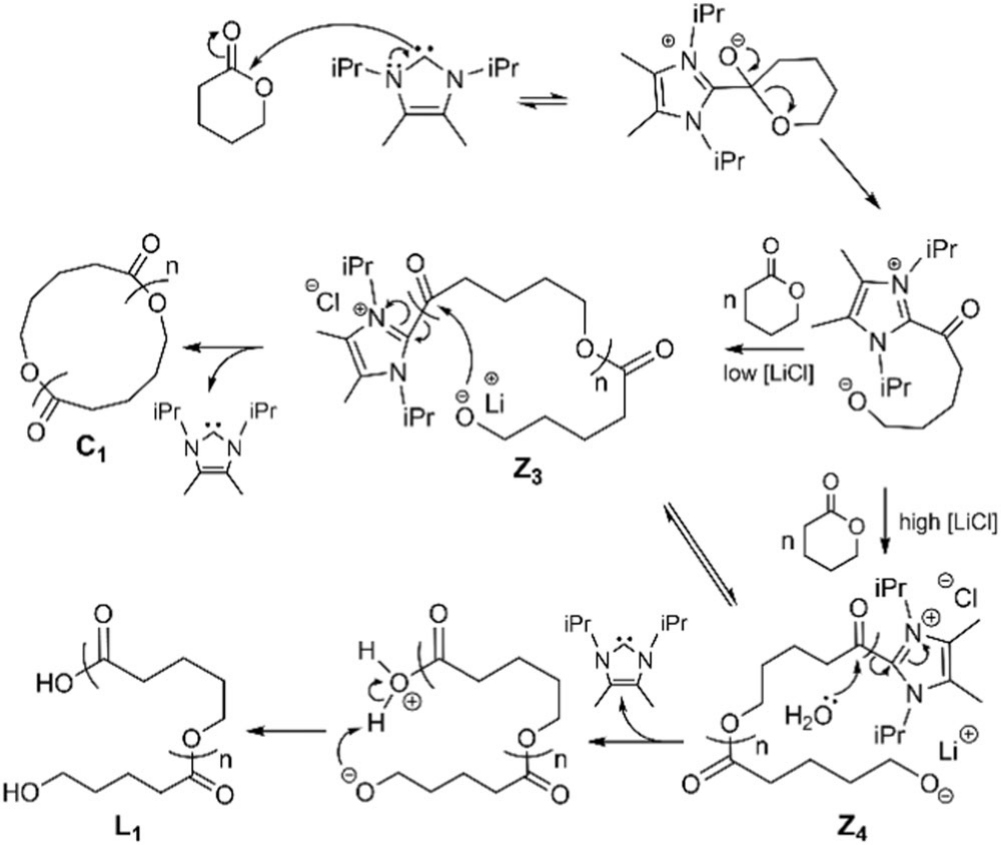
Ion pair effect on zwitterionic ring‐expansion polymerization. Adapted from Chang and Waymouth (2015) with RSC's permission.

**Figure 3:**
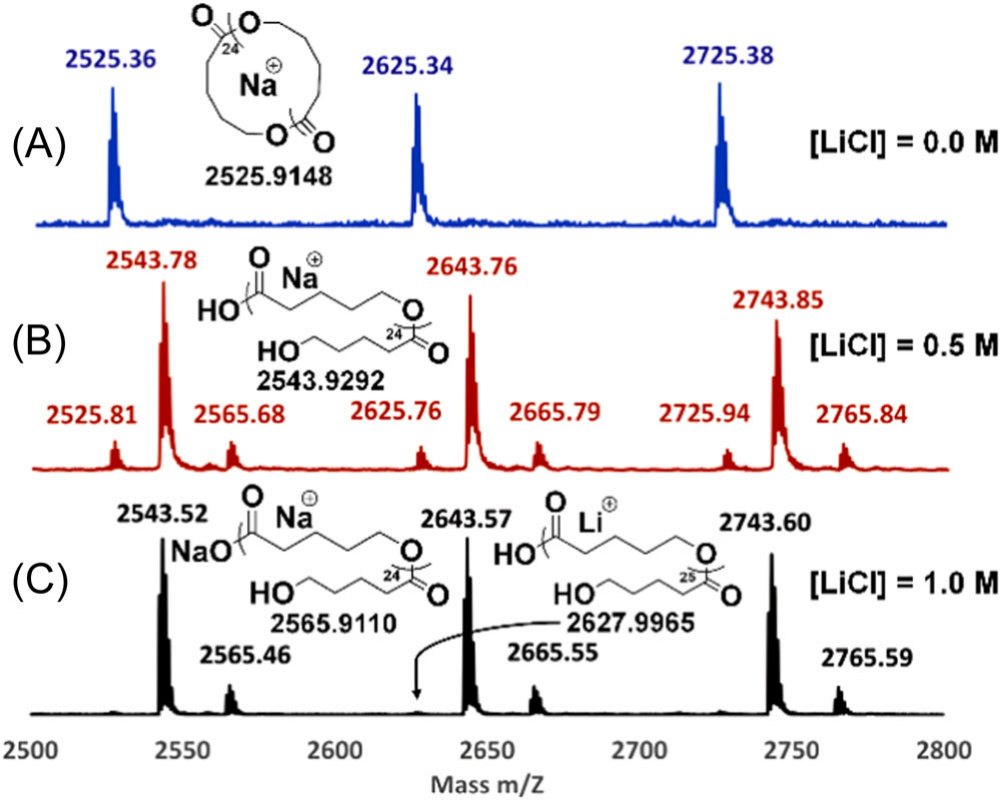
Matrix‐assisted laser desorption/ionization‐mass spectrometry spectra for poly(VL) at different concentration of LiCl. (A) [LiCl] = 0.0 M, (B) [LiCl] = 0.5 M, (C) [LiCl] = 1.0 M. Adapted from Chang and Waymouth (2015) with RSC's permission. [Color figure can be viewed at wileyonlinelibrary.com]

Chang et al. investigated the ion pairing effect on ZREP of cyclic poly(δ‐valerolactone) [c‐PVL)] in the presence of NHC‐3 (Scheme 4) by adding different concentrations of LiCl (Chang & Waymouth, 2015). In the absence of LiCl, cyclic polyester having MWs ranging from 16 to 39 kDa (by SEC) were achieved with dispersity varying from 1.2 to 1.5. Those authors proposed a mechanism showing the effect of Li^+^ and Cl^− ^in the ZREP starting from the initiation step to propagating step, which is evident by MALDI‐MS (Scheme 6). MALDI‐MS spectra display that the product was exclusively cyclic (**C**_**1**_ + Na^+^) when there was no LiCl present (Figure 3A), but with the addition of 0.5 M LiCl, these were mostly linear polymers (**L**_**1**_ + Na^+^ and **L**_**1**_ + H^+ ^+ 2Na^+^) with only a minor cyclic polymer peak (**C**_**1**_ + Na^+^) (Figure 3B). Finally, in the presence of 1.0 M LiCl, only linear polymers (**L**_**1**_ + Li^+^, **L**_**1**_ + Na^+^ and **L**_**1**_‐H^+^+2Na^+^) were generated (Figure 3C). In the latter condition, chlorine anion was attached to the imidazolium end of propagating zwitterion (Z_*n*_), and Li^+^ was attached to the alkoxide end, which converts into the linear polymer by the addition of water during the workup with aqueous methanol (Scheme 6). Furthermore, in the case of 0.5 M LiCl, the authors predict an equilibrium between the association and dissociation of LiCl between the zwitterions (**Z**_**3**_ and **Z**_**4**_); as a result, both the linear and cyclic products were formed.

To synthesize high MW cyclic polymer, cyclic polycaprolactone (ε‐PCL) was polymerized via ZREP at room temperature while using three different substituted NHCs (NHC‐1, NHC‐2, and NHC‐3) (Scheme 4) (Shin, Jeong, et al., 2011). According to SEC, they achieved MWs from 41 to 114 kDa with dispersity (Đ) 1.36–2.16. Moreover, the X‐ray scattering study shows that the crystallization rate of the cyclic polymer was faster than that of the analogs linear polymer.

**Scheme 7:**
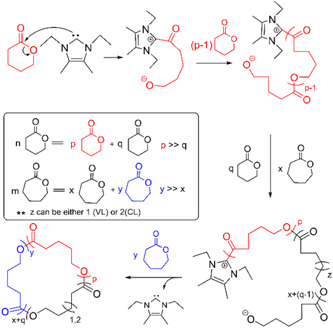
Zwitterionic ring‐expansion polymerization of gradient copolymer from δ‐VL & ε‐CL. Adapted from Shin, Brown, et al. (2011) with Wiley's permission. [Color figure can be viewed at wileyonlinelibrary.com]

**Figure 4:**
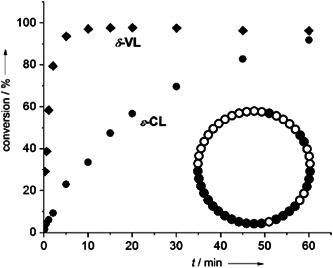
Conversion versus time graph for δ‐VL & ε‐CL during copolymerization. Adapted from Shin, Brown, et al. (2011) with Wiley's permission.

Waymouth and coworkers also synthesize cyclic gradient copolymer by ZREP from δ‐VL and ε‐CL using NHC‐2 (Scheme 4). Shin, Jeong, et al. (2011) explored this catalyst at room temperature to investigate the topological impact on molecular properties. For synthesizing gradient polymers, the two chosen monomers must demonstrate chain propagation with a substantial reactivity difference under the same reaction conditions (Matyjaszewski et al., 2000; Zaremski et al., 2009). According to SEC, they achieved gradient copolymers having MWs of 46–77 kDa with a dispersity of 1.7–2.4. The lower intrinsic viscosities of these obtained copolymers compared to their linear analogs confirmed the formation of the cyclic copolymer (Scheme 7). Furthermore, the studies of the percent conversion versus time shows that δ‐VL converts much faster than ε‐CL, which implies the formation of gradient copolymer (Figure 4). Moreover, the cyclic and linear gradient copolymers show lower melting points (MPs) than the analogous linear homopolymers of δ‐VL & ε‐CL and higher MPs than the analogous linear random copolymers. Similar NHC‐mediated synthesis of c‐PCL from ε‐CL and copolymer from δ‐VL & ε‐CL were also done by Vishwa and Yinghuai (2013).

**Scheme 8:**
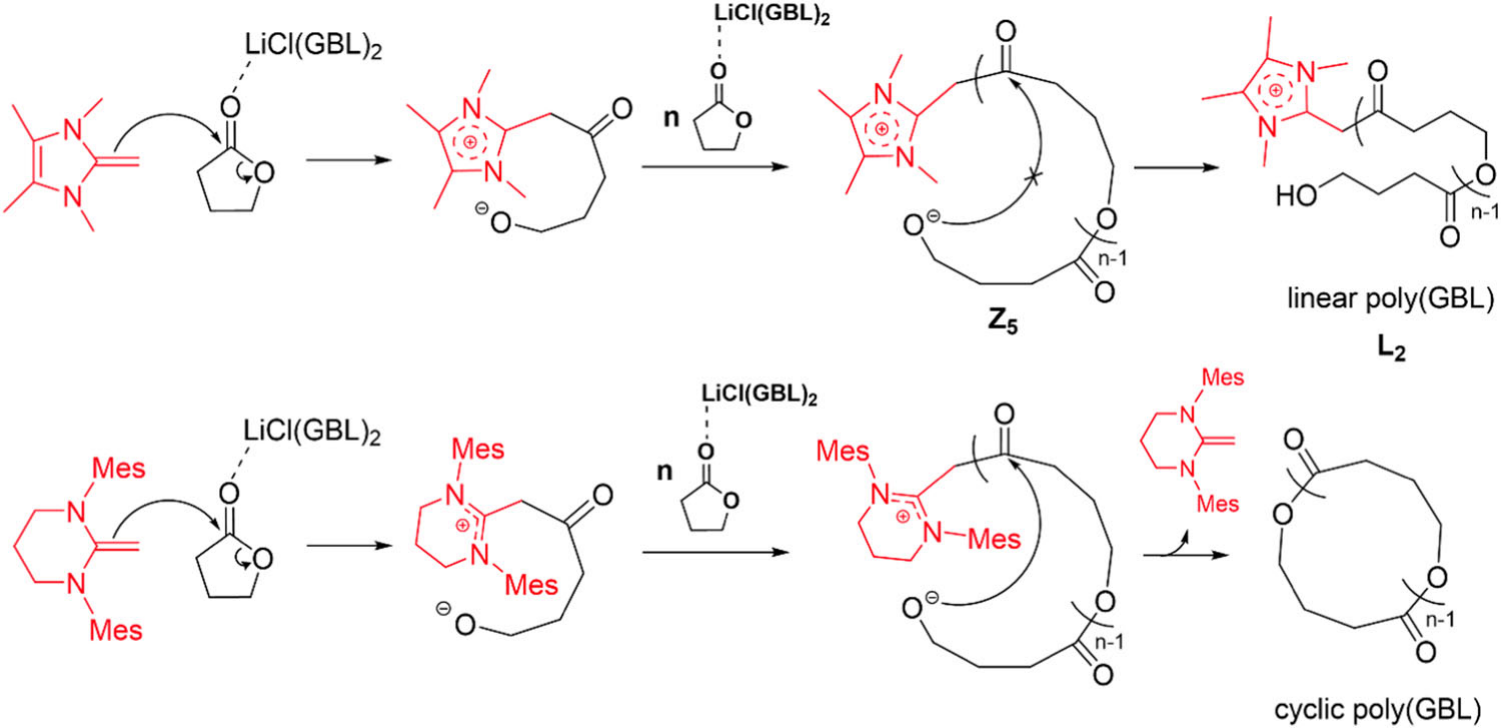
Zwitterionic ring‐expansion polymerization of Linear and cyclic poly(GBL) mediated by *N*‐heterocyclic olefin. Adapted from Walther et al. (2018) with ACS's permission. [Color figure can be viewed at wileyonlinelibrary.com]

**Figure 5:**
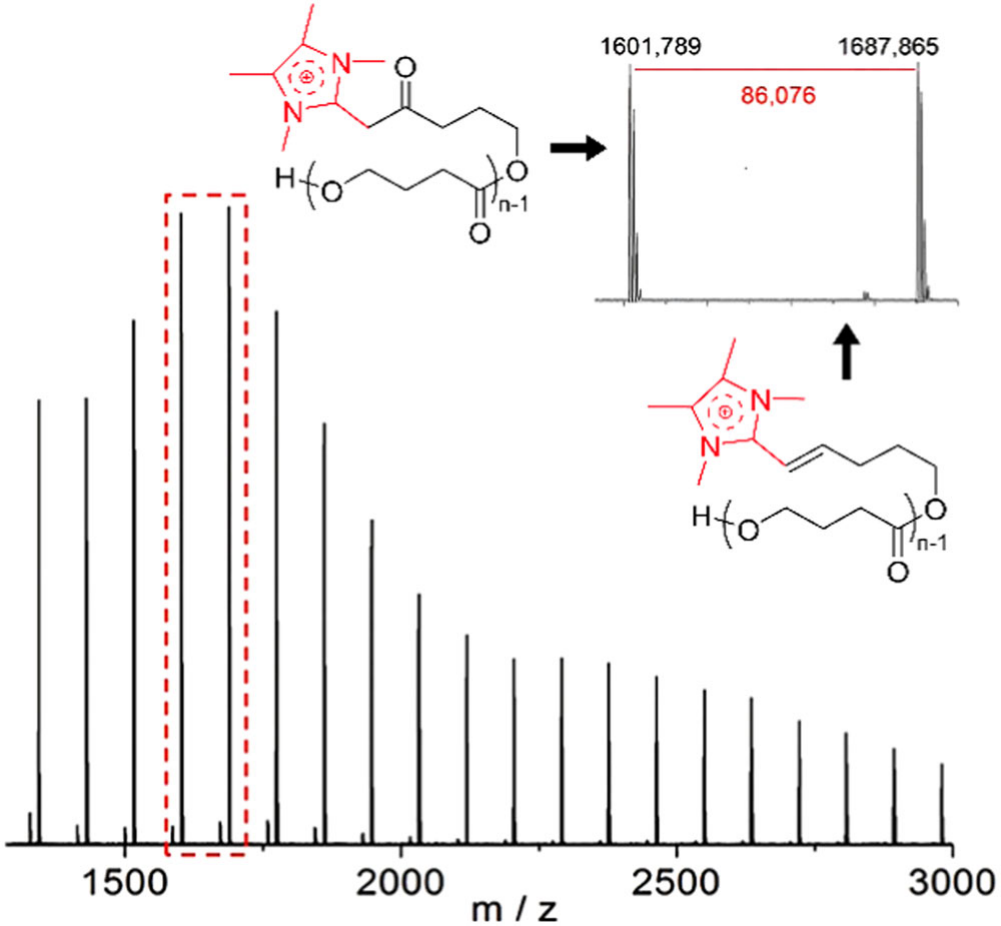
Matrix‐assisted laser desorption/ionization‐mass spectrometry spectrum of poly(GBL) mediated by *N*‐heterocyclic olefin. Adapted from Walther et al. (2018) with ACS's permission. [Color figure can be viewed at wileyonlinelibrary.com]

**Figure 6:**
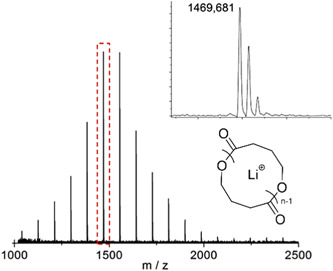
Matrix‐assisted laser desorption/ionization‐mass spectrometry spectrum of cyclic poly(GBL) mediated by *N*‐heterocyclic olefin. Adapted from Walther et al. (2018) with ACS's permission. [Color figure can be viewed at wileyonlinelibrary.com]

γ‐Butyrolactone (GBL) was polymerized by Walther et al. (2018) via a zwitterionic polymerization mechanism while using different *N*‐heterocyclic olefins (NHOs) as catalysts in the presence of lithium salt. On the one hand, without an alcohol initiator only NHO and GBL did not form any polymer; on the other hand, with an alcohol initiator no cyclic product was formed. To polymerize GBL without an alcohol initiator, LiCl was added to the NHOs, which (i) created a coordination bond with lactone, (ii) selectively activated the monomer, and (iii) also prevented the transesterification reactions (Scheme 8). However, linear and cyclic products were formed based on the nucleophilicity of the NHOs. Highly nucleophilic NHO‐1 (Scheme 4) generates linear product **L**_**2**_ by stabilizing zwitterionic chain **Z**_**5**_, which is evident by the MALDI‐MS spectrum (Figure 5). The very low‐intensity distribution along with **Z**_**5**_ in the MALDI‐MS spectrum could be from deoxygenation of the ketone (Figure 5). However, in the presence of the less nucleophilic NHO‐2 (Scheme 4), exclusively cyclic products (by MALDI‐MS and NMR) were formed as the catalyst stabilized the zwitterion very slightly (Figure 6), although the conversion was very low (6%). MW range of the cyclic products was 1000–2500 Da (by MALDI‐MS). This study shows a direct contrast between NHC and NHO‐mediated ZREP since NHC‐catalyzed polymerization without the initiator always generates cyclic products (Fastnacht et al., 2016) whereas different NHO produce different products.

### 2.2 ZREP of lactide

Waymouth and coworkers have also contributed to the field of ZREP of cyclic polyesters from lactide. In three of their studies (Brown et al., 2012; Culkin et al., 2007; Zhang & Waymouth, 2014) polyesters were obtained from lactide by utilizing three different catalysts: NHC, DBU (1,8‐diazabicyclo[5.4.0]undec‐7‐ene), and bicyclic isothioureas 1 & 2 (ITU 1 & 2) (Scheme 4). All those studies will be discussed below.

**Figure 7:**
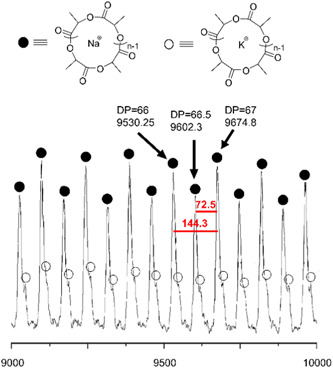
Matrix‐assisted laser desorption/ionization‐mass spectrometry spectrum of *N*‐heterocyclic carbenes mediated polylactide. Adapted from Shin, Brown, et al. (2011) with Wiley's permission. [Color figure can be viewed at wileyonlinelibrary.com]

Culkin et al. (2007) synthesized cyclic polylactide (PLA) from lactide monomer via ZREP in the presence of NHC‐4 (IMes) (Scheme 4) as the catalyst at room temperature. The cyclic structure was confirmed by MALDI‐MS, SEC, NMR, and viscosity measurement. PLA with MWs 7–26 kDa and a dispersity less than 1.4 were achieved according to SEC. MALDI‐MS spectrum shows that the product below 10 kDa was purely cyclic. In addition, the 72‐mass unit difference between MALDI‐MS peaks refers to cyclic PLA formation with odd and even numbers of lactic acid repeating units (Figure 7). The mechanism of this reaction is similar to the NHC‐mediated cyclic poly(lactone). Moreover, the cyclic product shows lower intrinsic viscosity than its linear analogs, and the viscosity ratio between the cyclic and linear product also closely matches the theoretical value. Similar NHC‐mediated syntheses of PLA were also performed by Prasad et al (2012) and Si et al. (2018).

Waymouth and coworkers explore the effectiveness of DBU and bicyclic isothioureas 1 & 2 (ITU 1 & 2) as catalysts instead of NHC for synthesizing cyclic PLA from lactide. At first, Brown et al. (2012) attempted to synthesize cyclic PLA using DBU as the catalyst, but the pure cyclic polymer was not generated because of the deprotonation next to cationic end of the zwitterionic intermediate, which eventually protonated the anionic site, leads to the formation of a neutral compound. Kemo et al. (2016) also used DBU as the catalyst to polymerize glycolide. However, they only obtained cyclic polymers as minor products, which they claimed were generated via ZREP.

**Scheme 9:**
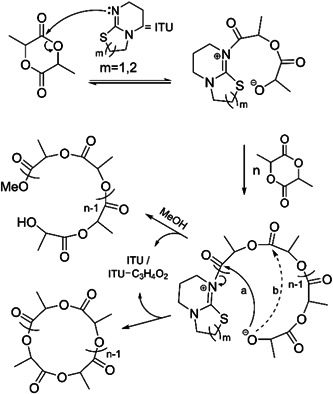
Zwitterionic ring‐expansion polymerization of polylactide using isothiourea as catalyst. Adapted from Brown et al. (2012) with ACS's permission.

**Figure 8:**
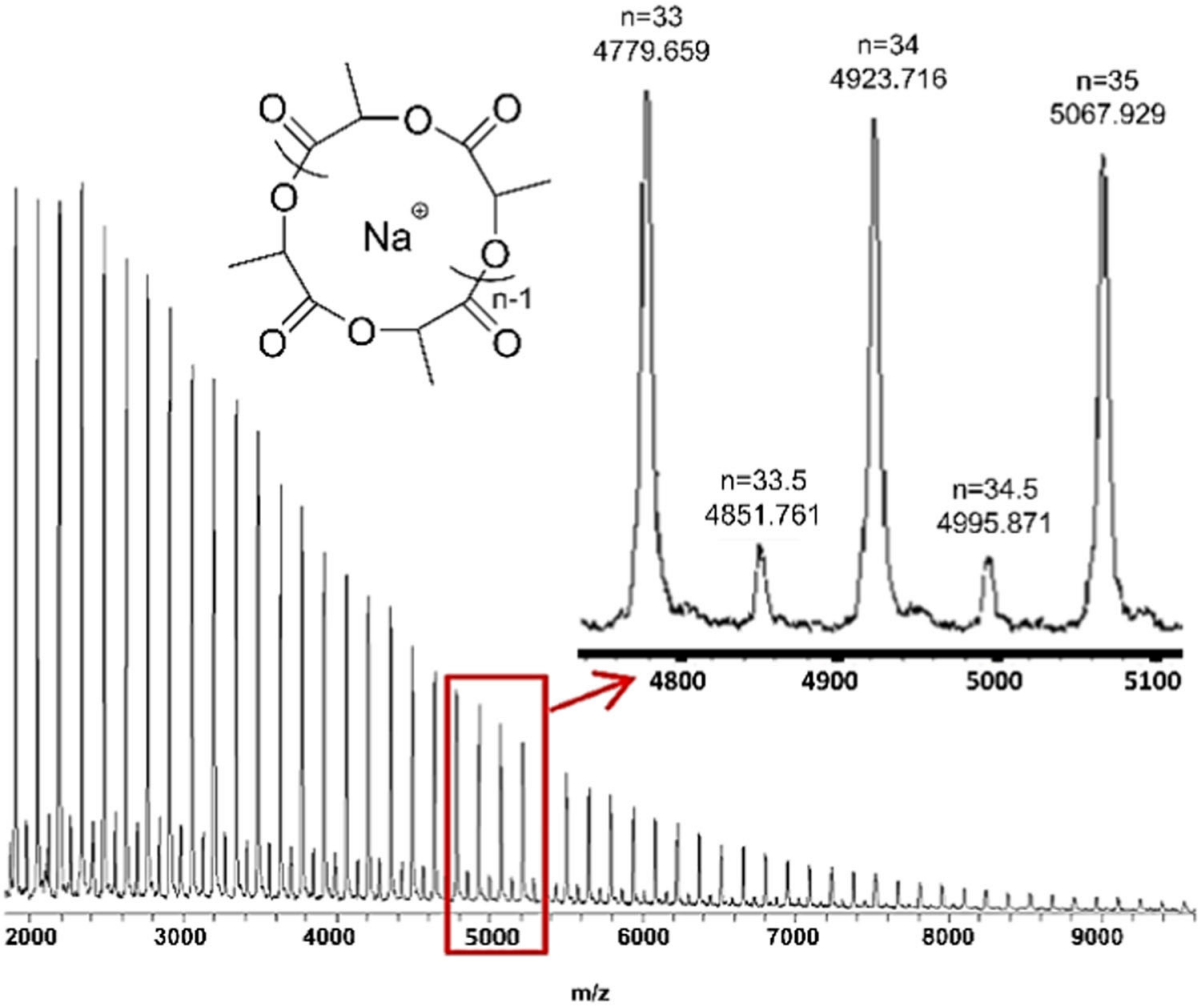
Matrix‐assisted laser desorption/ionization‐mass spectrometry spectrum of isothiourea mediated polylactide. Adapted from Brown et al. (2012) with ACS's permission. [Color figure can be viewed at wileyonlinelibrary.com]

Conversely, Zhang and Waymouth (2014) achieved almost pure cyclic PLA with narrow dispersity while using ITU 1 & 2 as the catalyst supported by MALDI‐MS and viscosity measurement. They also conducted mechanistic studies to compare the reactivity of ITU and DBU. In the case of viscosity measurement, the intrinsic viscosity ratio of [η]isothiourea/[η]linear = 0.68 was nearly the same as the theoretically projected (0.667) ratio for pure cyclic in a theta solvent (Fastnacht et al., 2016) (where the polymer chains behave as “ideal” chains). MALDI‐MS spectrum also claimed that the product was largely cyclic from 2 to 9 kDa. SEC defines the MWs range between 38 and 66 kDa. They have proposed a ZREP mechanism where cyclization of zwitterion can take place either as an attack on the carbonyl attached to the positive end of the zwitterion (path a) or backbiting on carbonyl (path b) (Scheme 9). The mechanism was supported by the presence of two distributions in the MALDI‐MS spectrum (Figure 8). The largest of these is the attack on the carbonyl adjacent to the positive end of the zwitterion which is around 80% of the MALDI‐MS data. However, the lower intensity are the peaks that give a 72 Da mass difference, which indicates the cyclic products have of the different pathways (path b) of cyclization (Figure 8). Moreover, to inspect why ITU generate macrocycles more selectively than DBU, they perform reactions between benzoyl chloride and the catalysts (ITU and DBU) separately in toluene. Both catalysts generate an acylated form, but DBU also produces a considerable amount of deprotonated compound, the source of linear impurities.

**Scheme 10:**
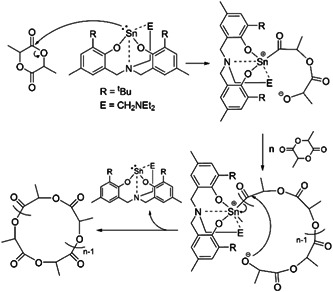
Zwitterionic ring‐expansion polymerization of polylactide using tin(II) complexes as catalyst. Adapted from Praban et al. (2019) with Wiley's permission.

**Figure 9:**
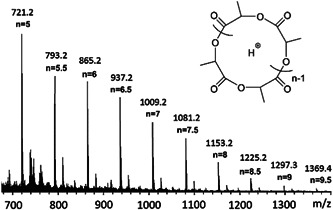
Matrix‐assisted laser desorption/ionization‐mass spectrometry spectrum of tin(II) complex‐mediated polylactide. Adapted from Praban et al. (2019) with Wiley's permission.

In 2019, bis(phenoxy)‐amine tin(II) complex (Scheme 4) were used as catalysts for ZREP of lactide to obtain cyclic PLA by Praban et al. (2019) Cyclic structure was confirmed by atmospheric pressure chemical ionization mass spectrometry (APCI‐MS), NMR, SEC, and intrinsic viscosity measurement. In the proposed mechanism, they claimed that the lone pair electron on Sn behaves the same way as carbene (NHCs) (Scheme 10). According to the APCI‐MS spectrum, the cyclic is the primary product, but APCI‐MS is not appropriate for MWs higher than 1500 Da (Holčapek et al., 2010) (Figure 9). However, the intrinsic viscosity versus MWs plot shows lower intrinsic viscosity of PLA synthesized in the absence of alcohol compared to the PLA obtained in the presence of alcohol, indicative of largely cyclic product formation aligning with earlier studies (Guo & Zhang, 2009, Jeong et al., 2009; Piedra‐Arroni et al., 2013).

Kricheldorf et al. (2008a) also synthesize cyclic PLA from lactide using imidazole and *N*‐methylimidazole (Scheme 4) as the catalyst. *N*‐methylimidazole‐catalyzed reaction follows ZREP, while imidazole‐catalyzed reaction undergoes anionic polymerization because of the *N*‐proton on imidazole. Based upon the MALDI‐MS data of *N*‐methylimidazole catalyzed PLA, cyclic product was present within MWs 1–7 kDa with several other mass peaks whose end groups are difficult to interpret. Cyclic PLA was less than 50% of the signals in the MALDI‐MS spectrum.

### 2.3 ZREP of other cyclic polyesters

Kricheldorf et al. (2008b) synthesize polyester from α‐hydroxyisobutyric acid anhydrosulfate (HiBAS) by implementing three different pathways (thermal, tertiary amine‐catalyzed, and alcohol‐initiated) where the thermal and the tertiary amine‐catalyzed generated cyclic polyester. According to the MALDI‐MS spectrum, the thermally (100^◦^C) prepared polymer has cyclic product (C) as major product until DP = 15 with seven smaller linear products. In higher degree of polymerization (DP) (after C_20_), the percentage of the cyclic product starts decreasing compared to linear, and eventually at DP = 42, cyclic is no longer the primary product. In the case of pyridine‐catalyzed synthesis, a mixture of cyclic and linear poly(HiBAS) was formed at 20^◦^C where the linear polymer is the primary product.

Cyclic poly(salicylide)s were synthesized through three reaction environments (thermal, carbene catalyzed, and imidazole initiated) from salicylic acid *O*‐carboxyanhydride (SOCA) by Kricheldorf et al. (2009) Among those three pathways, the carbene‐catalyzed reaction undergoes ZREP. In contrast, the thermal polymerization occurred by backbiting, and the imidazole‐catalyzed reaction follows anionic polymerization. According to the MALDI‐MS spectrum of the carbene‐catalyzed polymerization, the primary product was the cyclic poly(SOCA) with MWs between 1.2 and 4 kDa, with minor amounts of the linear product.

**Figure 10:**
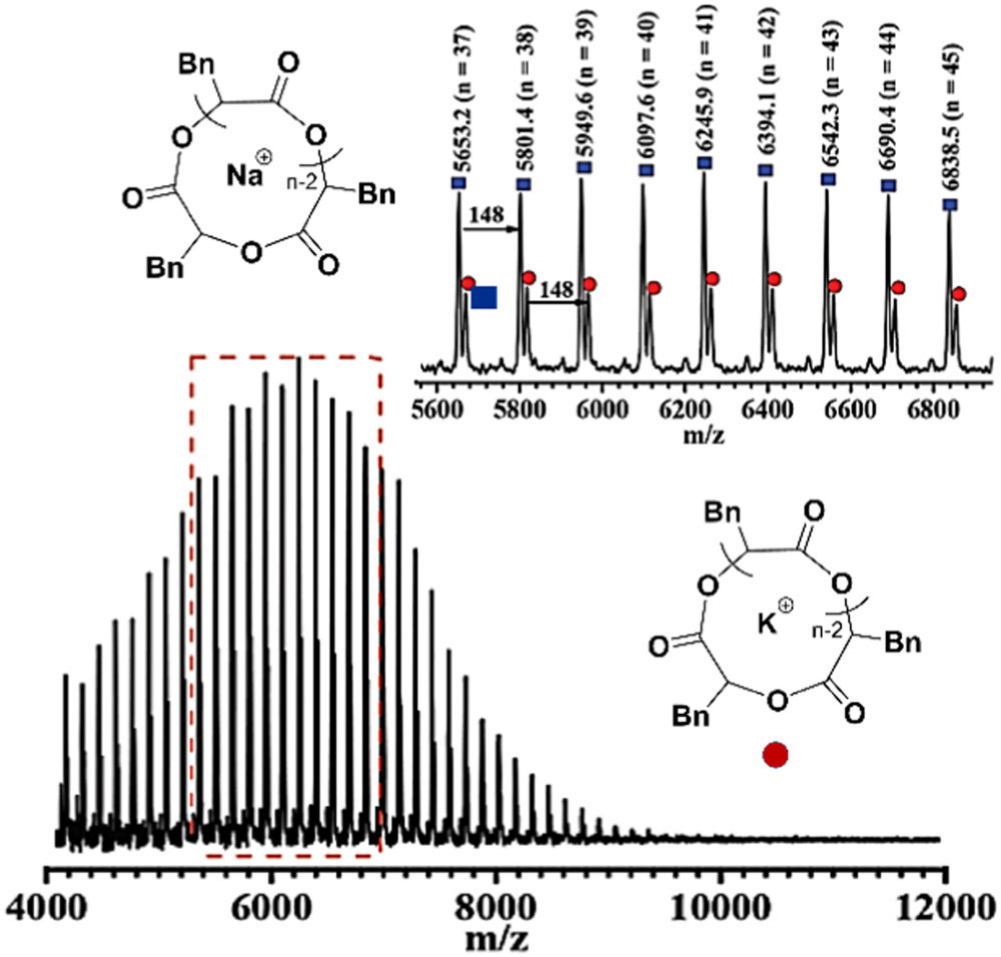
Matrix‐assisted laser desorption/ionization (MALDI)‐mass spectrometry spectrum of cyclic poly(PAHA) catalyzed by 1,4‐diazabicyclo‐[2.2.2]octane. Adapted from Liang et al. (2020) with RSC's permission. [Color figure can be viewed at wileyonlinelibrary.com]

**Scheme 11:**
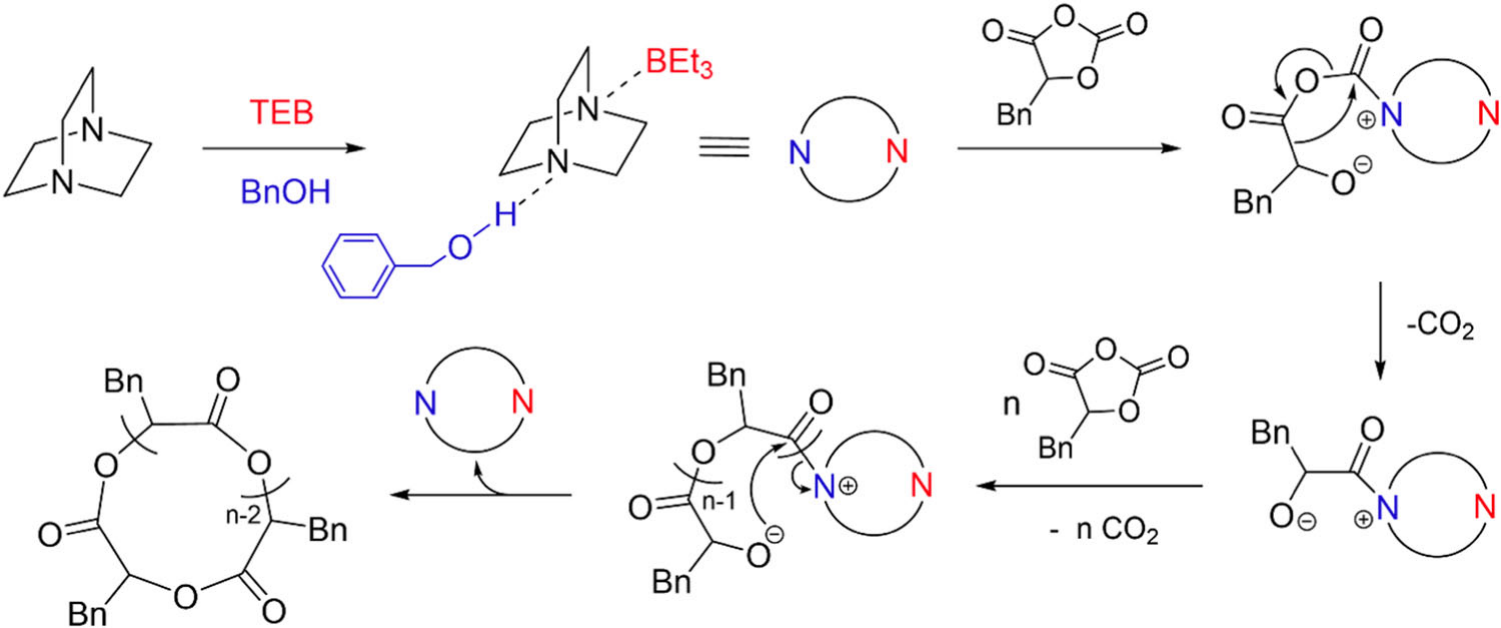
Zwitterionic ring‐expansion polymerization of cyclic poly(PAHA) catalyzed by 1,4‐diazabicyclo‐[2.2.2]octane. Adapted from Liang et al. (2020) with RSC's permission. [Color figure can be viewed at wileyonlinelibrary.com]

Liang et al. synthesized cyclic poly(α‐hydroxy acid)s (PAHAs) from *O*‐carboxyanhydrides (OCAs) via ZREP while using a combination of triethylborane (TEB), 1,4‐diazabicyclo‐[2.2.2]octane (DABCO) and benzyl alcohol (BnOH) (Scheme 4) as catalysts (Liang et al., 2020). This study was the first instance of ZREP with tertiary amine in the presence of alcohol. The authors claimed that the BnOH acted as a cocatalyst rather than the initiator. The cyclic structure was determined by MALDI‐MS (Figure 10), SEC, and intrinsic viscosity measurements. Although the authors claimed that no linear signal was observed, there is a lower intensity signal between the two cyclic signals, which could be emerging from BnOH terminated linear product. MALDI‐MS detected cyclic products within the MW range of 4–10 kDa. Moreover, the lower intrinsic viscosity of the product compared to the linear analogs also supports the cyclic topology. The proposed ZREP mechanism elucidated that DABCO becomes a single‐site catalyst by adding TEB on one side and BnOH on the other, making monomer activation easier to propagate the zwitterionic chain (Scheme 11).

**Figure 11:**
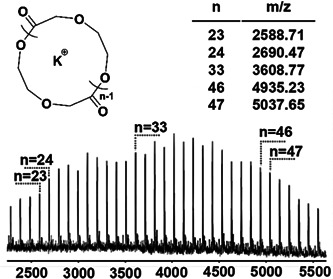
Matrix‐assisted laser desorption/ionization (MALDI)‐mass spectrometry spectrum of cyclic poly(ether ester). Adapted from Li et al. (2022) with Wiley's permission.

**Scheme 12:**
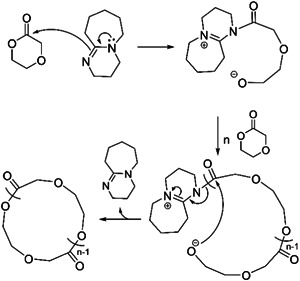
Mechanism of ZROP of cyclic poly(ether ester). Adapted from Li et al. (2022) with Wiley's permission.

Lastly, Li et al. (2022) synthesized a cyclic poly(ether ester) from *p*‐dioxanone (PDO) via ZREP while using DBU as the catalyst. In the presence of alcohol PDO produces a linear product, whereas in the absence of alcohol a cyclic product is formed. The cyclic topology was evident by ^1^H NMR, ^1^C NMR, and MALDI‐MS. According to the MALDI‐MS mass spectrum of the DBU‐mediated reaction in the absence of alcohol, the product was exclusively cyclic (Figure 11). MALDI‐MS detected cyclic products between 2.5 and 5.5 kDa. The proposed mechanism other than monomer was very similar to the ZREP of lactone by DBU (Scheme 12).

Out of all the catalysts used to synthesize cyclic polyester via ZREP, NHCs (Scheme 4) were the most successful in generating predominantly cyclic products compared to other catalysts: DBU, ITU, DABCO, NHOs, tin complex (Scheme 4). This can be attributed to the strong nucleophilicity of NHCs which accelerates polymerization initiation.

## 3 ZREP OF CYCLIC POLYAMIDE

**Figure 12:**
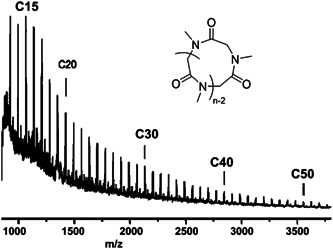
Matrix‐assisted laser desorption/ionization‐mass spectrometry spectrum of poly(Sar) with pyridine/*N*‐methylpyrrolidone. Adapted from Kricheldorf et al. (2006) with Wiley's permission.

**Scheme 13:**
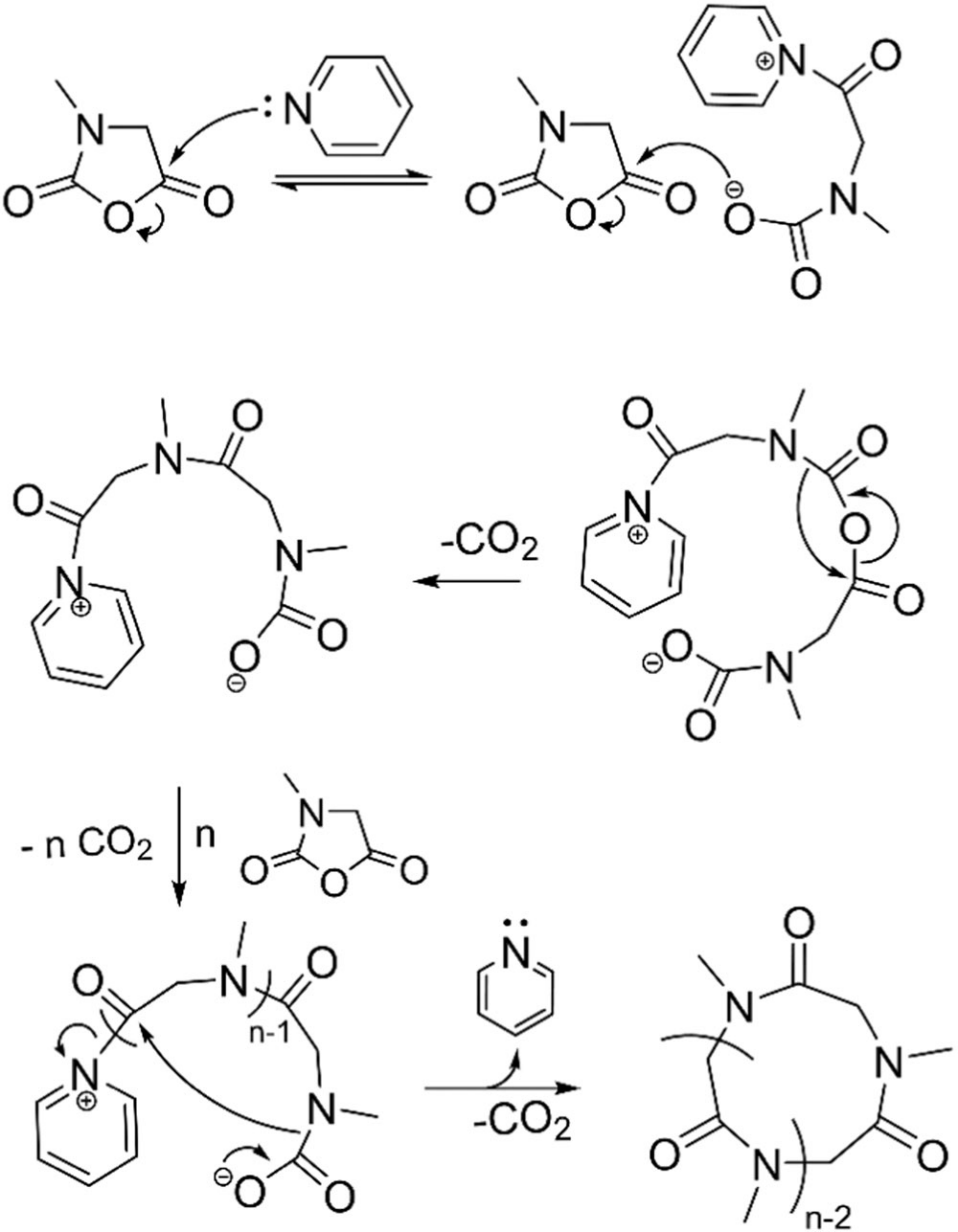
Pyridine catalyzed zwitterionic ring‐expansion polymerization of Sar‐NCA. Adapted from Kricheldorf et al. (2006) with Wiley's permission.

Kricheldorf et al. (2006) synthesize cyclic polyamide via ZREP in 2006. In this study, four different α‐amino acid *N*‐carboxyanhydride (NCA) (sarcosine NCA [Sar‐NCA], d,l‐phenylalanine NCA [d,l‐Phe‐NCA], d,l‐alanine NCA, and d,l‐leucine NCA) were polymerized using pyridine, *N*‐ethyldiisopropylamine (EDPA) and *N,N*‐dimethylaminopyridine (DMAP) (Scheme 4) as the catalyst in three different reaction medias (pyridine, *N*‐methylpyrrolidone [NMP], and dioxane). Among all those different combinations of reactions, only Sar‐NCA and d,l‐Phe‐NCA generate cyclic polypeptoids as the main products. In the case of Sar‐NCA, the reaction in neat pyridine, where pyridine acts as both the reaction medium and the catalyst, produced a cyclic polyamide as the primary product with moderate linear mixtures. Furthermore, the same reaction was performed in NMP as reaction medium and pyridine as catalyst (1:1 Sar‐NCA/pyridine), generating primarily cyclic polymer with some minor linear products (Figure 12). According to a previous study by Kricheldorf et al. (2005), the absence of cyclic oligomers (in the pyridine‐initiated polymerization) verified the zwitterionic mechanism where lone pair electrons of pyridine nitrogen attack the carbonyl group of NCA (Scheme 13). Besides, NMP promotes zwitterionic polymerization because of its polarity when present as the solvent. By MALDI‐MS, cyclic polyamide is the primary product between 800 and 3000 Da (Figure 12), but after 3500 Da, the linear overwhelms the cyclic. On the other hand, although d,l‐Phe‐NCA produces cyclic products with EDPA in dioxane, it is not a ZREP since the lone‐pair electron cannot attack the NCA carbon to generate a zwitterionic intermediate due to the steric hindrance of EDPA (Scheme 4).

Zhang and coworkers have reported at least four different cyclic polyamides and two diblock copolyamides via ZREP (Guo & Zhang, 2009; Guo et al., 2011; Lahasky et al., 2011; Lee et al., 2011; Li et al., 2016). They have used five *N*‐substituted *N*‐carboxyanhydrides (NCA) [^*N*^methyl NCA, ^*N*^butyl NCA, (S)/(R)‐^*N*^(1‐phenylethyl) NCA, ^*N*^propargyl NCA, ^*N*^decyl NCA] as the monomer while using a particular NHC named 1,3‐bis(2,6‐diisopropylphenyl)imidazolidin‐2‐ylidene) [SIPr] for all but one study as the catalyst (Scheme 4). These studies are discussed in the following section.

**Scheme 14:**
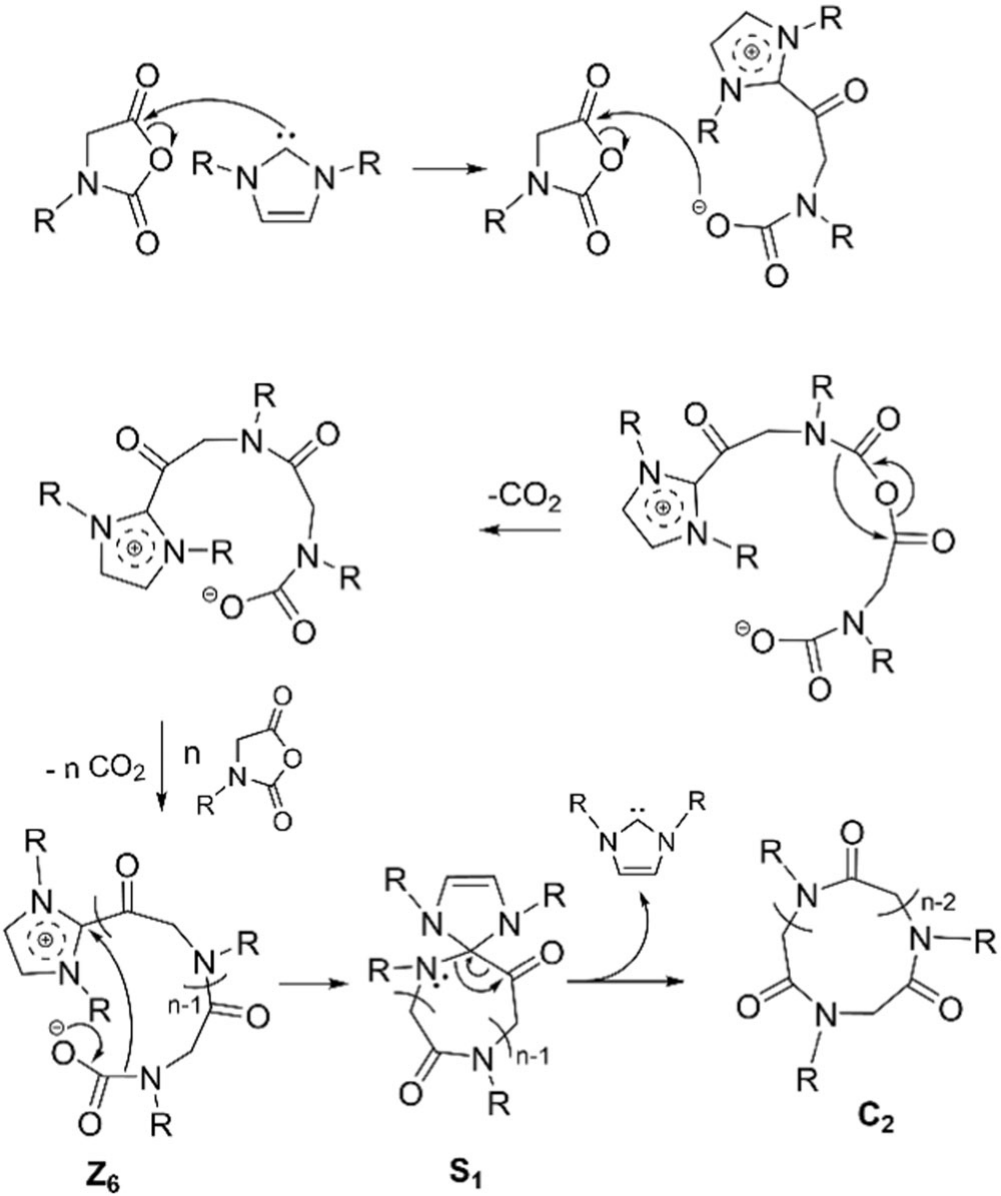
Zwitterionic ring‐expansion polymerization of cyclic poly(^*N*^Me‐glycine) and poly(^*N*^Bu‐glycine). Adapted from Guo and Zhang (2009) with ACS's permission.

**Figure 13:**
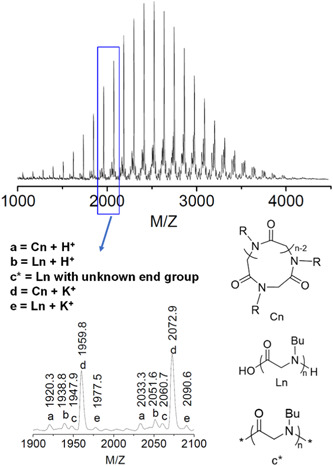
Matrix‐assisted laser desorption/ionization‐mass spectrometry spectrum of poly(^*N*^Bu‐glycine). Adapted from Guo and Zhang (2009) with ACS's permission. [Color figure can be viewed at wileyonlinelibrary.com]

Guo and Zhang polymerized ^*N*^methyl NCA [^*N*^Me‐NCA] and ^*N*^butyl NCA [^*N*^Bu‐NCA] to obtain cyclic poly(^*N*^Me‐glycine) and cyclic poly(^*N*^Bu‐glycine) in the presence of SIPr (NHC‐5, Scheme 4) as the catalyst via ZREP (Scheme 14) (Guo & Zhang, 2009). As evidence for the formation of **C**_**2**_ (Scheme 14), SEC characterization of cyclic poly(^*N*^Bu‐glycine)s (NHC mediated) shows lower intrinsic viscosities compared to the linear analogs (amine mediated) having the same MWs. Several reactions were performed with different monomer‐to‐initiator ratios to investigate the control over MWs. The experimental MWs were similar to the theoretical MWs between the 3 and 11 kDa (by SEC). They also claimed that a lower concentration of zwitterions (**S**_**1**_ rather than **Z**_**6**_ in the equilibrium) reduces the side reactions. To bolster their claim, they conducted the polymerization reaction in the presence of dimethyl formamide, which can stabilize the zwitterions. As a result, they lost control over MWs because more side reactions occurred due to the high concentration of zwitterions. Moreover, the MALDI‐MS spectrum of poly(^*N*^Bu‐glycine) exhibits primarily the cyclic products (a, b, c) with a small amount of linear products (d, e) (Figure 13). Furthermore, to generate cyclic poly(^*N*^Me‐glycine)‐*b*‐poly(^*N*^Bu‐glycine), ^*N*^Me‐NCA, and ^*N*^Bu‐NCA monomers were added subsequently. SEC spectra confirmed the formation of the copolymer, and the absence of poly(^*N*^Me‐NCA) in the final product indicates the living polymerization for the first monomer (^*N*^Me‐NCA). Additional evidence in regard to copolymer formation is provided by NMR. Using the same approach, they also synthesized cyclic poly(^*N*^1‐phenylethyl glycine) (Guo et al., 2011), cyclic poly(N‐propargyl‐ glycine) (Lahasky et al., 2011), cyclic poly(^*N*^decyl‐ glycine) (Lee et al., 2011), and cyclic poly(^*N*^Me‐glycine)‐b‐poly(^*N*^decyl‐glycine) (Lee et al., 2011).

**Scheme 15:**
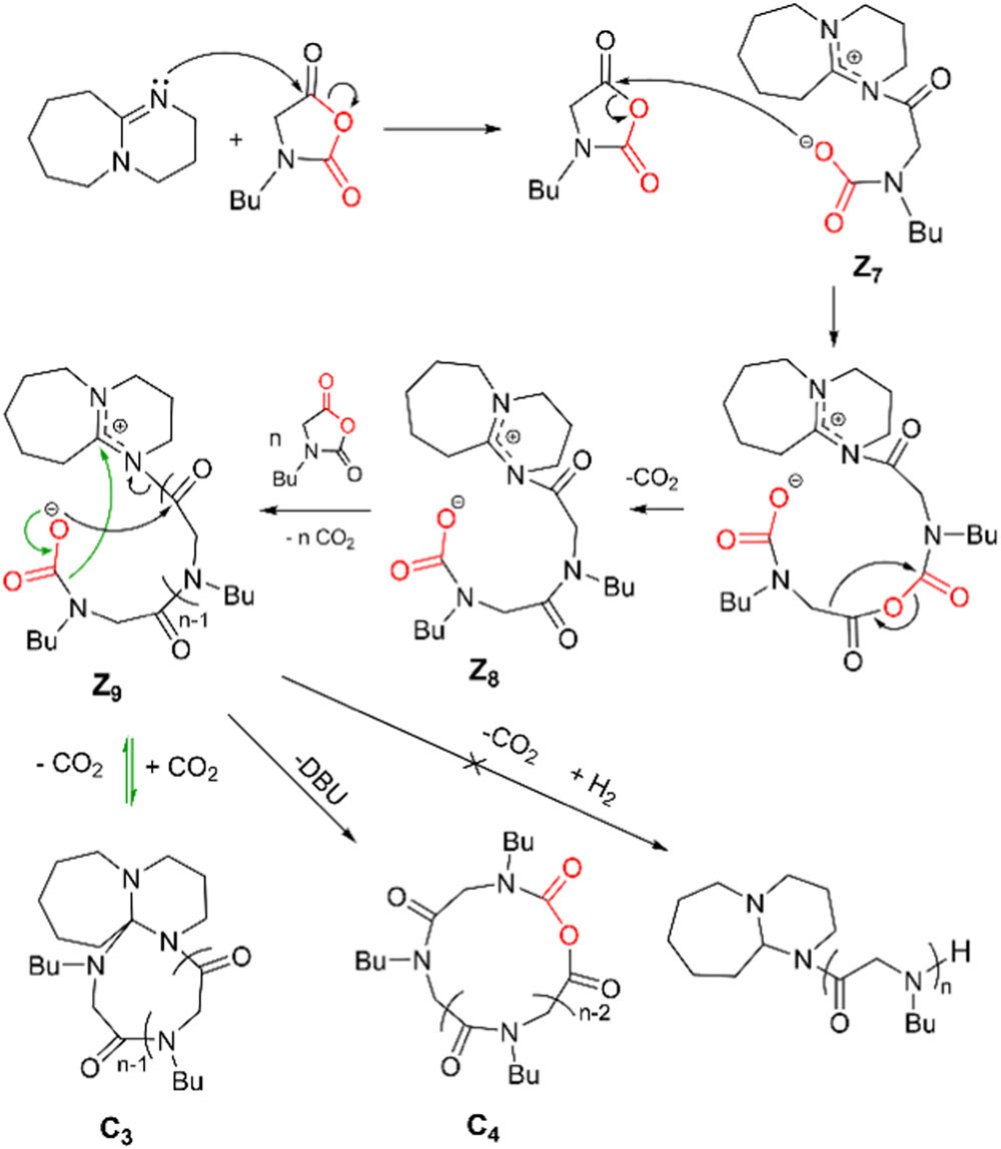
Mechanism of zwitterionic ring‐expansion polymerization of poly(^*N*^Bu‐glycine). Adapted from Li et al. (2016) with ACS's permission. [Color figure can be viewed at wileyonlinelibrary.com]

**Figure 14:**
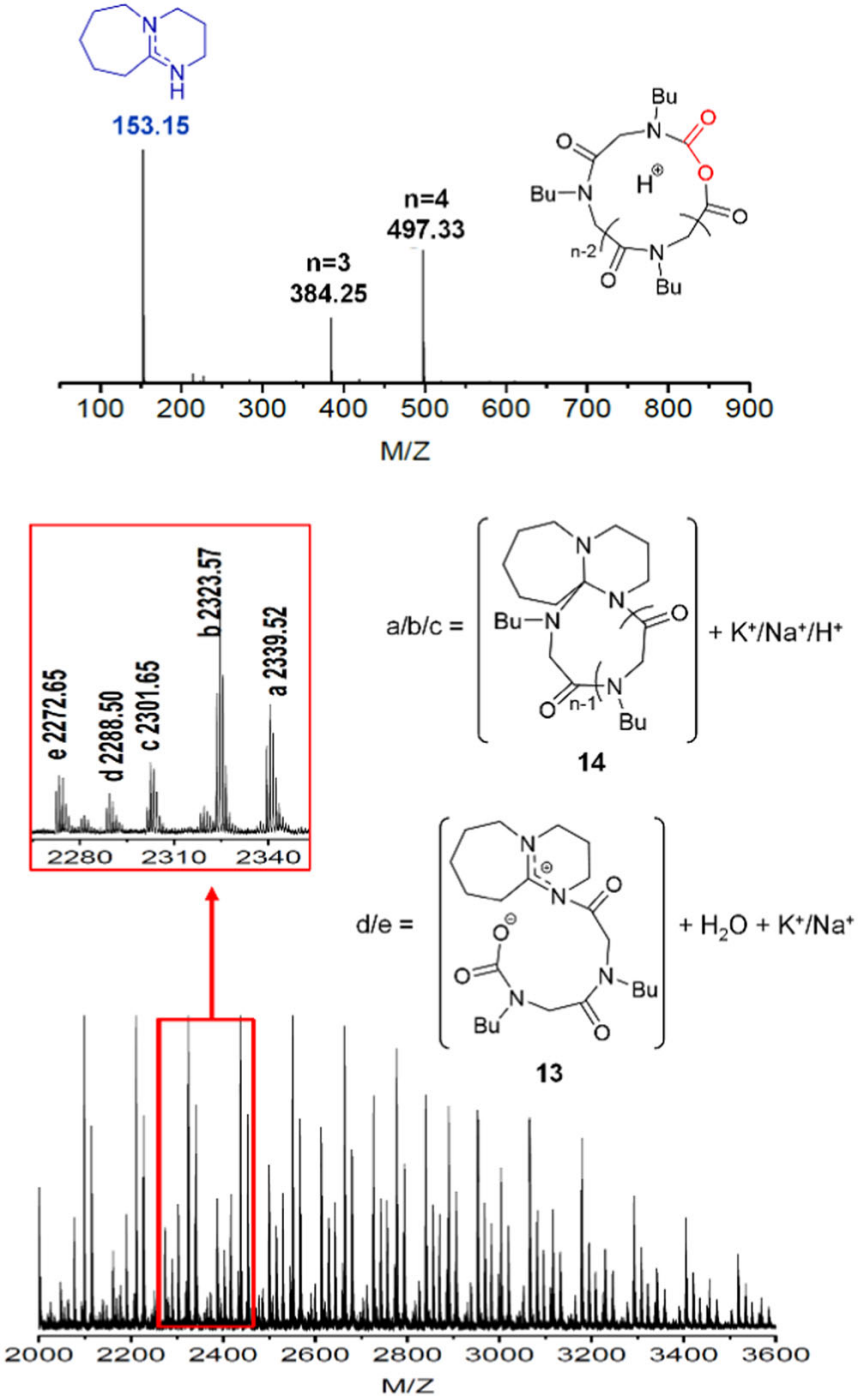
(Bottom) matrix‐assisted laser desorption/ionization‐mass spectrometry spectrum of lower molecular weight poly(^*N*^Bu‐glycine), (Top) electrospray ionization mass spectrometry spectrum while monomer:initiator = 1:1. Adapted from Li et al. (2016) with ACS's permission. [Color figure can be viewed at wileyonlinelibrary.com]

Li et al. also synthesize cyclic poly(^*N*^Bu‐glycine) from ^*N*^Bu‐NCA via ZREP using DBU (Scheme 4) instead of NHC as the catalyst (Li et al., 2016). By removing the air and moisture‐sensitivity sensitivity of NHC catalysts, DBU has increased the control over MWs. The nitrogen lone pair from DBU attacked the carbonyl group to obtain **Z**_**7**_, which will propagate with more monomers attacking the carboxylate group generating **Z**_**8**_ (Scheme 15). MALDI‐MS spectrum of lower MW (bottom, Figure 14) shows the presence of zwitterionic propagating chains **Z**_**9**_ and cyclic poly(^*N*^Bu‐glycine) with one DBU unit **C**_**3**_ (Scheme 15). The formation of **Z**_**9**_ from **Z**_**8**_ through removing the CO_2_ group is supported by spectroscopic evidence provided in previous studies (Dell'Amico et al., 1986; Rawlinson & Humke, 1972) which again undergo another decarboxylation to form cyclic **C**_**3**_, evident by MALDI‐MS (bottom, Figure 14). Additionally, the ESI‐MS spectrum proves the elimination of DBU to form **C**_**4**_ from **Z**_**9**_ (top, Figure 14). By MALDI‐MS, the cyclic polymer is the primary product as **C**_**3**_, presents in four different forms (a, b, c) with only a small amount of linear compounds (d, e) (Figure 14). According to SEC, MWs with a wide range (3.5–32.4 kDa) were achieved by controlling the monomer‐to‐catalyst ratio. Moreover, kinetic studies show that DBU‐catalyzed polymerization (Walther et al., 2018) has a slightly faster propagation rate than previous NHC‐catalyzed polymerizations (Brown et al., 2012; Culkin et al., 2007; Fastnacht et al., 2016; Vishwa & Yinghuai, 2013). One possible explanation the authors proposed is the difference in steric effect between DBU and NHC‐initiated zwitterionic intermediates **Z**_**9**_ and **Z**_**6**_.

Unlike cyclic polyester, few works have been done on synthesizing cyclic polyamide via ZREP. Successful ZREP of cyclic polyamide depends on both the suitable monomer and the initiator. In the case of catalysts, NHCs showed promising results, whereas DBU and pyridine‐catalyzed syntheses have previously produced impure cyclic products (Table 1).

**Table 1:** Concise summary of polymers, monomers, catalysts, analytical techniques, and major conclusions discussed in this review.

Polymers	Monomer type	Monomers	Catalysts	Analytical techniques	Conclusion	References
Polyesters	Lactones	β‐PL	SIMes	– ESI‐MS – SEC – ^1^H NMR	– Primarily cyclic (by ESI‐MS) – MW: 6 kDa (by SEC) – Dispersity: 1.16 (by SEC)	Jeong et al. (2007)
		β‐BL	SIMes	– ESI‐MS – SEC – ^1^H NMR	– mixture of cyclic and linear (by ESI‐ MS) – MW: 5 kDa (by SEC) – Dispersity: 1.42 (by SEC)	Jeong et al. (2007)
		δ‐VL	NHC‐3	– MALDI‐MS – SEC – ^1^H NMR	– Primarily cyclic (by MALDI‐MS) – MW range: 16–39 kDa (by SEC) – Dispersity: 1.2–1.5 (by SEC)	Chang and Waymouth (2015)
		ε‐CL	NHC‐1, NHC‐2, & NHC‐3	– SEC – ^1^H NMR – Viscometry	– Pure cyclic (by ^1^H‐NMR) – MW range: 41–114 kDa (by SEC) – Dispersity: 1.36–2.16 (by SEC)	Shin, Brown, et al. (2011)
		δ‐VL & ε‐CL	NHC‐2	– SEC – ^1^H NMR – Viscometry	– Pure cyclic (by intrinsic viscosity measurement) – MW range: 46–77 kDa (by SEC) – Dispersity: 1.7–2.4 (by SEC)	Shin, Jeong, et al. (2011)
		γ‐BL	NHO‐2	– MALDI‐MS – ^1^H NMR	– Primarily cyclic (by MALDI‐MS & ^1^H NMR) – MW: 1–2.5 kDa (by MALDI‐MS)	Walther et al. (2018)
	Lactide		NHC‐4	– MALDI‐MS – SEC – ^1^H NMR – Viscometry	– Pure cyclic (All) – MW range: 7–26 kDa (by SEC) – Dispersity: <1.4 (by SEC)	Culkin et al. (2007)
			DBU	– MALDI‐MS – SEC – ^1^H NMR	– Mixture of cyclic and linear (by MALDI‐MS) – MW: 8 kDa (by SEC) – Dispersity: 1.19 (by SEC)	Zhang and Waymouth (2014)
			ITU 1 & 2	– MALDI‐MS – SEC – ^1^H NMR – Viscometry	– Mixture of cyclic and linear (by MALDI‐MS & intrinsic viscosity measurement) – MW range: 38–66 kDa (by SEC) – Dispersity: 1.39–1.65 (by SEC)	Brown et al. (2012)
			NHCs	– APCI‐MS – SEC – ^1^H NMR – Viscometry	– Primarily cyclic (by APCI‐MS & intrinsic viscosity measurement) – MW: 9 kDa (by SEC) – Dispersity: 1.28 (by SEC)	Praban et al. (2019)
			Imidazole & NMI	– MALDI‐MS – ^1^H NMR	– Mixture of cyclic and linear (by MALDI‐MS) – MW: 1–6 kDa (by MALDI‐MS)	Kricheldorf et al. (2008a)
	Others	HiBAS	Heat & TEA	– MALDI‐MS – SEC – ^1^H NMR	– mixture of cyclic and linear (by MALDI‐MS) – MW: 2–9 kDa (by SEC) – Dispersity: 1.25–2.8 (by SEC)	Kricheldorf et al. (2008b)
		SOCA	Triazolyl carbene	– MALDI‐MS – ^1^H NMR	– Primarily cyclic (by MALDI‐MS) – MW: 1.2–4 kDa (by MALDI‐MS)	Kricheldorf et al. (2009)
		OCAs	TEB, DABCO, & BnOH	– MALDI‐MS – SEC – ^1^H NMR – Viscometry	– Pure cyclic (by MALDI‐MS & intrinsic viscosity measurement) – MW range: 3–47 kDa (by SEC) – Dispersity: 1.13–1.33 (by SEC)	Liang et al. (2020)
		PDO	DBU	– MALDI‐MS – SEC – ^1^H NMR	– Pure cyclic (by MALDI‐MS) – MW: 8 kDa (by SEC) – Dispersity: 1.1 (by SEC)	Li et al. (2022)
		Cyclic anhydrides	BF_3_ & quinoline	– MALDI‐MS – ^1^H NMR	– Mixture of cyclic and linear (by MALDI‐MS & ^1^H NMR)	Kricheldorf and Petermann (2001)
Polyamides	Sar‐NCA		Pyridine	– MALDI‐MS – ^1^H NMR	– Mixture of cyclic and linear (by MALDI‐MS)	Kricheldorf et al. (2006)
	^*N*^Bu‐glycine		SIPr	– MALDI‐MS – SEC – ^1^H NMR	– Primarily cyclic (by MALDI‐MS) – MW: 8 kDa (by SEC) – Dispersity: 1.04–1.12 (by SEC)	Guo and Zhang (2009)
	^*N*^Bu‐NCA		DBU	– MALDI‐MS – ESI‐MS – SEC – ^1^H NMR	– Primarily cyclic (by MALDI‐MS, and ESI‐MS) – MW range: 3.5–32.4 kDa (by SEC) – Dispersity: 1.02–1.12 (by SEC)	Li et al. (2016)
Polyethers	GPE		B(C_6_F_5_)_3_	– MALDI‐MS – SEC – ^1^H NMR	– Mixture of cyclic and linear (by MALDI‐MS) – MW range: 1–12 kDa (by SEC) – Dispersity: 1.5–1.9 (by SEC)	Asenjo‐Sanz et al. (2014)
	THF & GPE			– MALDI‐MS – SEC – ^1^H NMR	– Mixture of cyclic and linear (by MALDI‐MS) – MW range: 30–175 kDa (by SEC) – Dispersity: 1.6–1.8 (by SEC)	Asenjo‐Sanz et al. (2015)
	PGE, Cl‐PGE, BGE, ETD, ECH, SO, F‐SO, Cl‐SO			– MALDI‐MS – SEC – ^1^H NMR	– Mixture of cyclic and linear (by MALDI‐MS) – MW range: 1–26 kDa (by SEC) – Dispersity: 1.3–2.6 (by SEC)	Haque et al. (2019)
	glycidol			– MALDI‐MS – SEC – ^1^H NMR	– Mixture of cyclic and linear (by MALDI‐MS) – MW range: 3–8 kDa (by SEC) – Dispersity: 1.3–4 (by SEC)	Al Assiri et al. (2022)
Poly‐carbonates	8CC_Bn_, 8CC_Ph_		NHC‐3	– MALDI‐MS – SEC – ^1^H NMR	– Pure cyclic by MALDI‐MS) – MW range: 14–96 kDa (by SEC) – Dispersity: 1.61–1.92 (by SEC)	Chang et al. (2016)
	TMC		TBD	– MALDI‐MS – SEC – ^1^H NMR	– Pure cyclic by MALDI‐MS) – MW range: 14–33 kDa (by SEC) – Dispersity: 1.19–1.55 (by SEC)	Azemar et al. (2021)
Polysiloxanes	TMOSC		NHC‐1, NHC‐3, & NHC‐4	– MALDI‐MS – SEC – ^1^H NMR	– Pure cyclic by MALDI‐MS) – MW range: 27–86 kDa (NHC‐4), 587 kDa (NHC‐3), and 942 kDa (NHC‐1) [by SEC] – Dispersity: 1.39–3.18 (by SEC)	Brown et al. (2013)
Poly(alkylene phosphates)	iPP		NHC‐1 & NHC‐3	– MALDI‐MS – ^1^H NMR – Light scattering (LS)	– Primarily cyclic by (MALDI‐MS) – MW range: 11–202 kDa (by LS) – Dispersity: 1.05–1.44 (by LS)	Stukenbroeker et al. (2014)

## 4 ZREP OF CYCLIC POLYETHERS

**Figure 15:**
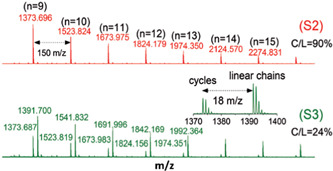
Matrix‐assisted laser desorption/ionization‐mass spectrometry spectra of poly(GPE) in anhydrous (S2)and aqueous (S3) medium. Adapted from Asenjo‐Sanz et al. (2014) with RSC's permission. [Color figure can be viewed at wileyonlinelibrary.com]

Asenjo‐Sanz and coworkers synthesize cyclic polyether via ZREP (Asenjo‐Sanz et al., 2014) while using tris(pentafluorophenyl)borane [B(C_6_F_5_)_3_] (Scheme 4) as the catalyst at room temperature. Asenjo‐Sanz et al. described the ZREP mechanism of the monomer, glycidyl phenyl ether (GPE) to yield cyclic poly(GPE). In addition to operating the reaction both in anhydrous and aqueous systems, they also did DFT calculations in support of the proposed mechanism. By MALDI‐MS, the reaction without water yielded 90% C/L (eventually some of these cyclic were found to be tadpoles), while in the presence of water, C/L is decreased to 24%. This is evidence for the formation of the zwitterionic intermediate because the reaction between the water and intermediate can produce a higher percentage of the linear product by eliminating B(C_6_F_5_)_3_ (Figure 15).

**Scheme 16:**
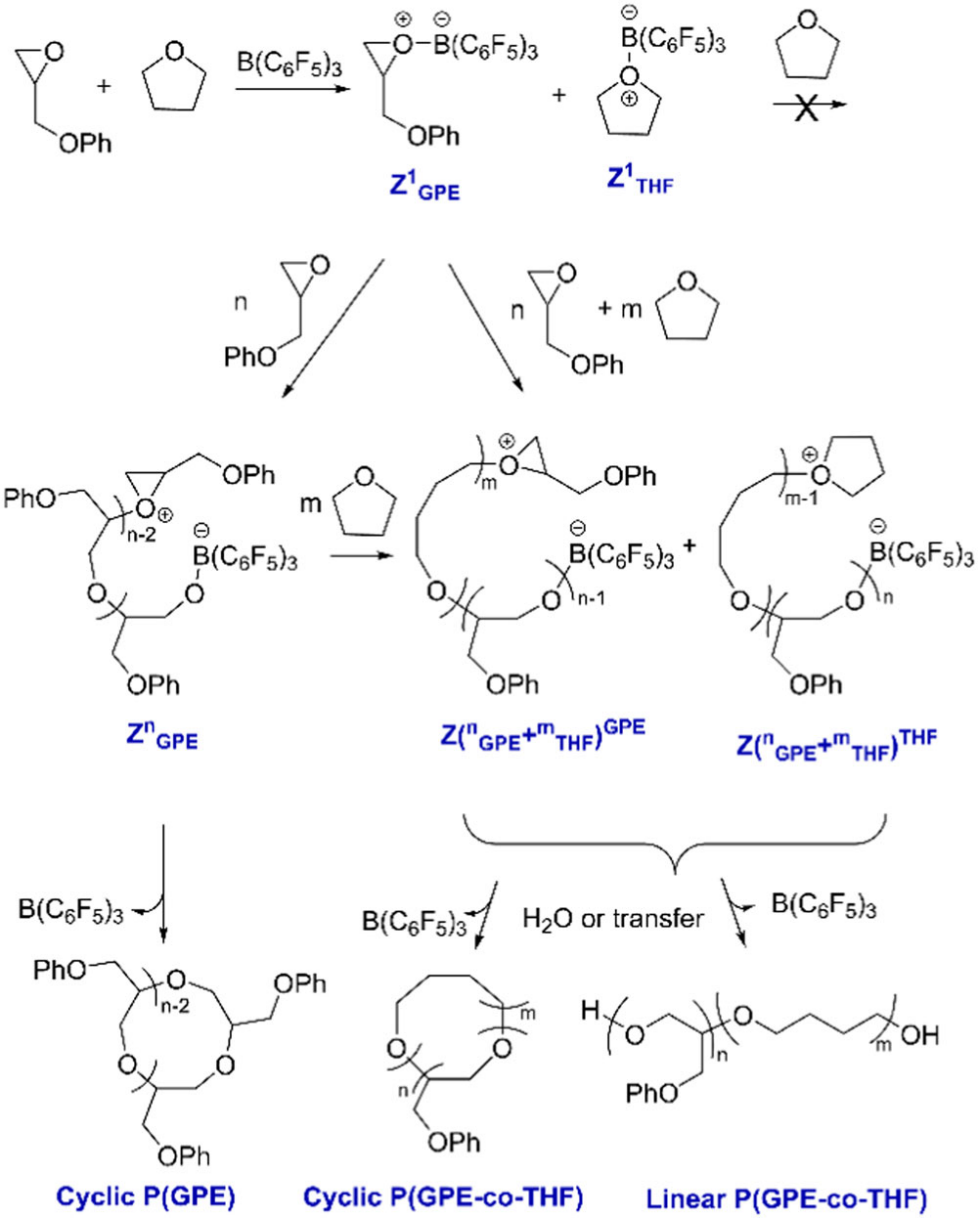
Mechanism of zwitterionic ring‐expansion polymerization of poly(GPE‐co‐THF). Adapted from Asenjo‐Sanz et al. (2015) with ACS's permission. [Color figure can be viewed at wileyonlinelibrary.com]

**Figure 16:**
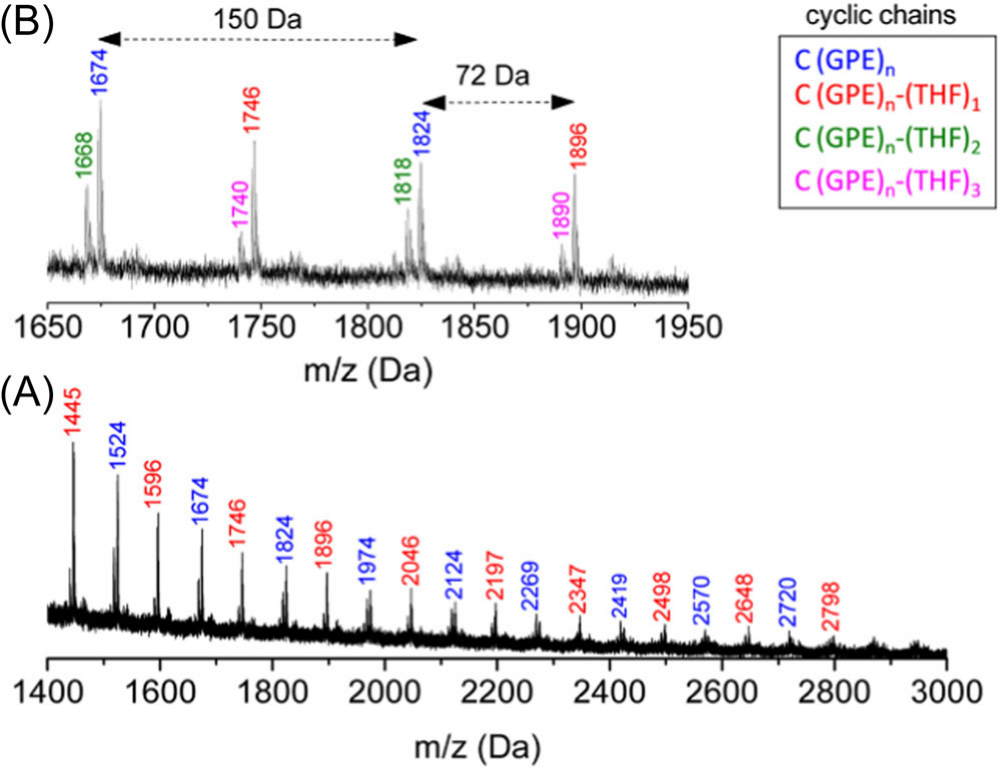
Matrix‐assisted laser desorption/ionization‐mass spectrometry spectra of (A) poly(GPE‐co‐THF) when GPE]^0^/[THF]^0^ = 80/20 mol %, (b) zoomed in from 1650 to 1950 Da. Adapted from Asenjo‐Sanz et al. (2015) with ACS's permission. [Color figure can be viewed at wileyonlinelibrary.com]

Asenjo‐Sanz et al., in their second investigation associated with ZREP, reported several polymerization reactions between the different ratios of tetrahydrofuran (THF) and glycidyl phenyl ether (GPE) at room temperature to obtain copolymers [poly(GPE‐co‐THF)] while using the same Lewis acid [B(C_6_F_5_)_3_] catalyst (Asenjo‐Sanz et al., 2015). The proposed ZREP mechanism (Scheme 16) was supported by MALDI‐MS spectra and DFT calculations. From the series of reactions, they observed that above 78 mol% of THF fraction (in the mixture of THF and GPE) produces a linear copolymer as a major product. Furthermore, if a large excess of GPE is polymerized (in the mixture of THF and GPE), both cyclic poly(GPE‐co‐THF) and cyclic(PGE) were observed. The absence of a cyclic homopolymer with just THF indicates Z^1^_GPE_ as the propagating zwitterion, which is also supported by DFT calculation. DFT calculations show the ΔH_298.15_ value for Z^1^_GPE_ (−10.43 kcal/mol) is higher than Z^1^_THF_ (−13.91 kcal/mol), indicating that the less stable and more highly reactive Z^1^_GPE_ can yield more cyclic polymer. The presence of cyclic(PGE) in MALDI‐MS data supports the formation of Z^n^_GPE_. The MW of the cyclic copolymer is from 200 to 3000 Da when [GPE]°/[THF]° = 80/20 mol% by MALDI‐MS (Figure 16). On the other hand, MWs of linear copolymer went up to 333 kDa with [GPE]°/[THF]^0^ = 2/98 mol % according to SEC.

**Scheme 17:**
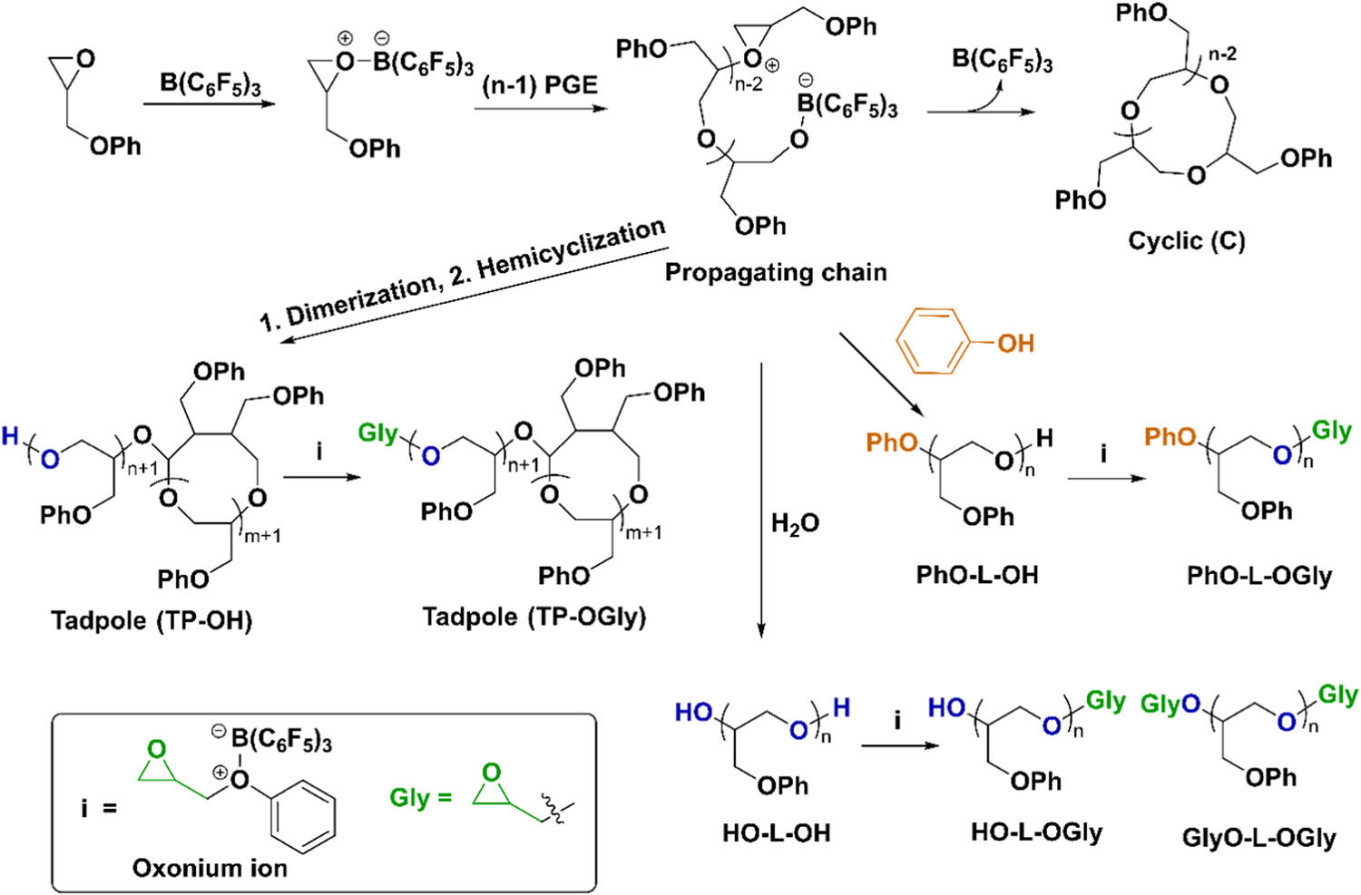
Mechanism for the different architecture formation during polymerization of Poly(PGE). Adapted from Haque et al. (2019) with ACS's permission. [Color figure can be viewed at wileyonlinelibrary.com]

Haque et al. (2019) synthesized macrocycle polyethers from monosubstituted epoxides via electrophilic ZREP in the presence of B(C_6_F_5_)_3_ as initiator at room temperature. In this project, to study the effect of substituent on polymerization, they investigated eight different monomers: three monomers from the glycidyl ether(GE) family (phenyl glycidyl ether [PGE], 4‐chlorophenyl glycidyl ether [Cl‐PGE], and benzyl glycidyl ether [BGE]), three from styrene oxide(SO) family (SO, 4‐chlorostyrene oxide [Cl‐SO], and 4‐fluorostyrene oxide [F‐SO]), epichlorohydrin (ECH), and 1,2‐epoxytetradecane (ETD). Several mechanisms were discussed based on the data obtained from the MALDI‐MS spectra for each monomer to figure out the origins of impurities which could have been very challenging to estimate using other conventional characterization techniques. One common impurity is the monohydroxylated tadpole polymer (TP‐OH) which has the same MW as cyclic polymer. This originated from the dimerization of propagating chain followed by hemicyclization (Scheme 17). The second well‐known impurity, dihydroxy linear polymer (HO − L − OH) is developed by a trace amount of water that adds to propagating chain. In the case of the glycidyl ether family, most of the other linear and tadpole impurities are generated due to the formation of the oxonium ion via the ether‐cleavage mechanism of epoxide monomers. However, most of these impurities can be removed by the “click scavenging” of all the hydroxyl‐containing impurities by attaching these to an azide (by MALDI‐MS) (Scheme 17). A substantial reduction of impurities in poly(Cl‐PGE) compared to poly(PGE) was observed as the electron‐withdrawing property of chlorine makes the *p*‐chlorophenol a weaker leaving group causing the inhibition of the ether cleavage mechanism through oxonium ion. Conversely, poly(BGE) produces more additional impurities because of the cationic reactivity of the benzylic position. Furthermore, in the case of monomers without glycidyl ether linkage (ETD, ECH, SO), fewer impurities were expected as the formation of oxonium ions via the ether‐cleavage mechanism was implausible. As a result, ETD generates a less contaminated cyclic polyether. However, ECH shows several impurities due to the formation of chloronium ion, and the SO family exhibits around 5% conversion due to deactivation by the [B(C_6_F_5_)_3_] initiator. The MW of the two polymers (Cl‐PGE and ETD) with less impurities are 2100 and 2600 Da respectively (by SEC).

**Figure 17:**
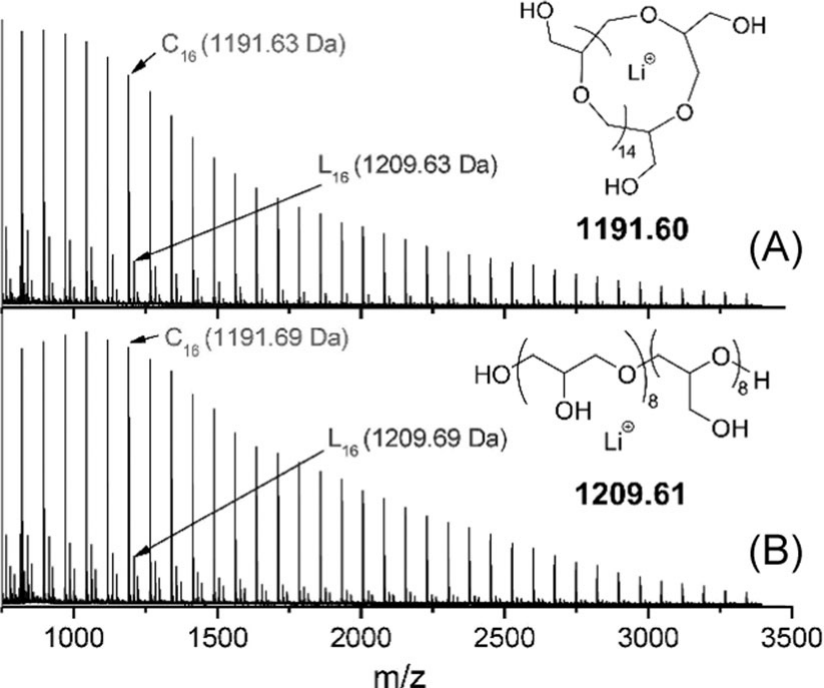
Matrix‐assisted laser desorption/ionization‐mass spectrometry spectra of polyglycidol in (A) absence (B) presence of molecular sieves. Adapted from Al Assiri et al. (2022) with Elsevier's permission.

**Scheme 18:**
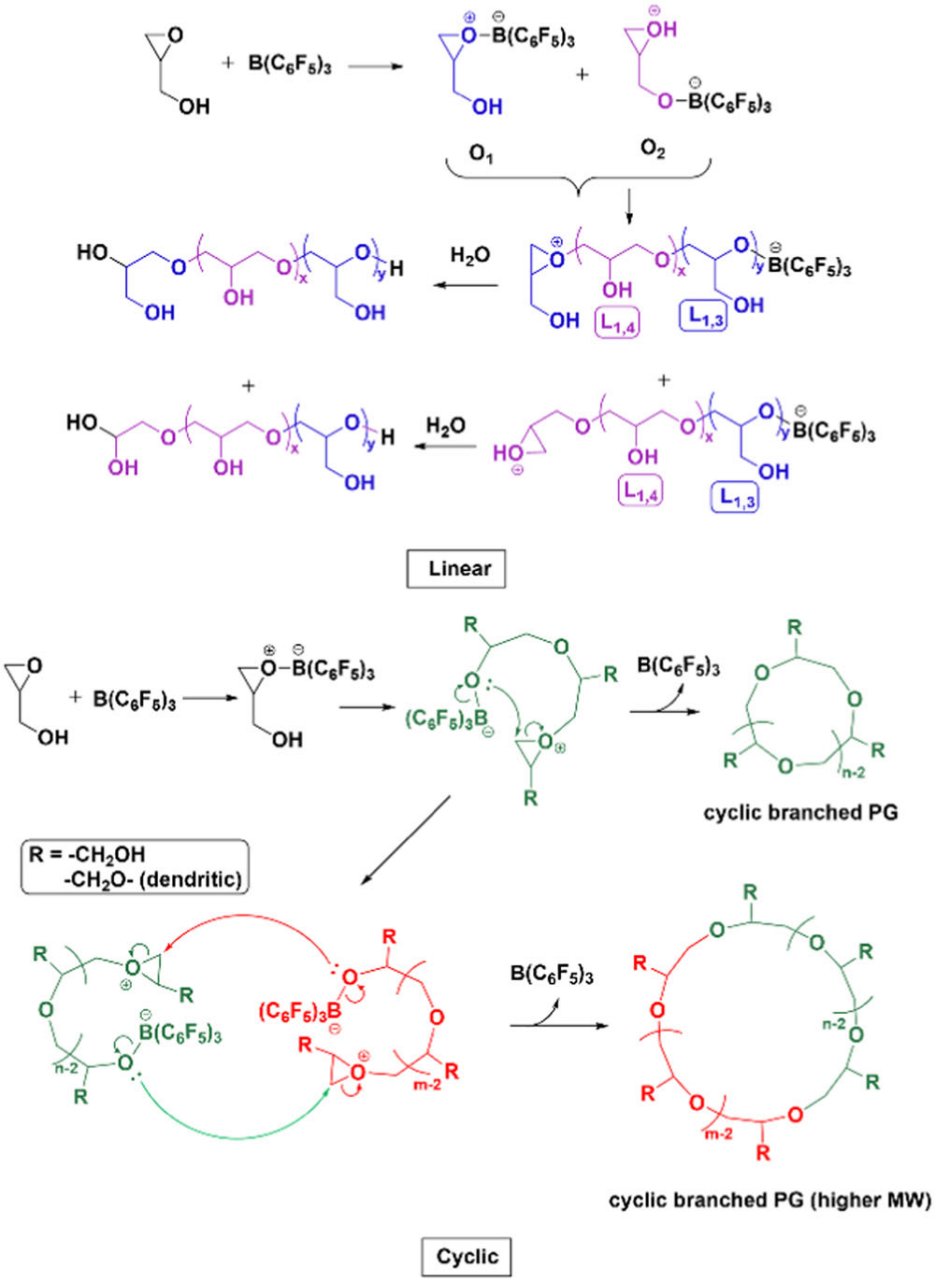
Mechanism of ZROP of polyglycidol. Adapted from Al Assiri et al. (2022) with Elsevier's permission. [Color figure can be viewed at wileyonlinelibrary.com]

Recently, Al Assiri et al. (2022) synthesized cyclic polyglycidol from glycidol while using tris(pentafluorophenyl)borane [B(C_6_F_5_)_3_] as the catalyst. Polymerization reactions were conducted with different monomer‐to‐catalyst ratios in the presence and absence of molecular sieves. Both cyclic and linear product formation were detected in the MALDI‐MS spectra regardless of the presence or absence of molecular sieves, indicating the sieve's ineffectiveness in removing water (Figure 17). Based on the MALDI‐MS data, the cyclic product was the primary product within MWs range 500–3500 kDa, along with the linear impurities. However, in some cases MALDI‐MS shows high‐intensity cyclic peaks because of higher ionization efficiency of cyclic polymer compared to the linear counterpart (Aubry et al., 2010; Weidner & Kricheldorf, 2020). Moreover, the proposed ZREP mechanism describes the electrophilic attack on B(C_6_F_5_)_3_ can take place by glycidol oxygens to form oxonium ions (**O**_**1**_ and **O**_**2**_) (Scheme 18). Both oxonium ions reacted with each other to form L_1,3_ and L_1,4_ units in propagating zwitterionic chains, according to the previous studies (Tokar et al., 1994). In addition, based on the mechanism of the earlier studies, two different mechanisms (intermolecular Asenjo‐Sanz et al., 2014) and intramolecular (Engler & Kohl, 2020; Kammiyada et al., 2013, 2016) ring closer mechanism) for termination to achieve the cyclic product were proposed (Scheme 18). The intermolecular and intramolecular ring‐closing mechanism yielded a cyclic product, whereas the addition of water results in the linear product with two hydroxyl ends (Scheme 18).

All the ZREP of cyclic polyether have used the same catalyst tris(pentafluorophenyl)borane [B(C_6_F_5_)_3_]. So far, the major drawback of B(C_6_F_5_)_3_ is the production of tadpoles, which have the same MWs as our desired cyclic products. O'Neill et al. (2022) show a successful separation and identification of those tadpole impurities from the cyclic analogs using ultrahigh performance liquid chromatography‐MS/MS (UHPLC‐MS/MS). Click scavenging is another way of getting rid of specific impurities (Haque et al., 2017). However, more investigation is required in this field to avoid chromatographic separation or complex scavenging routes.

## 5 ZREP OF OTHER CYCLIC POLYMERS

### 5.1 ZREP of cyclic polycarbonates

**Scheme 19:**
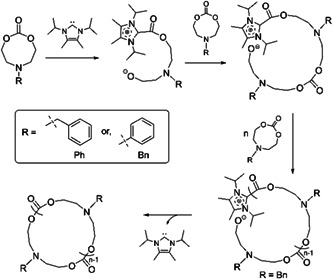
Zwitterionic ring‐expansion polymerization of eight‐membered cyclic carbonates with *N*‐heterocyclic carbenes catalyst. Adapted from Chang et al. (2016) with ACS's permission.

**Figure 18:**
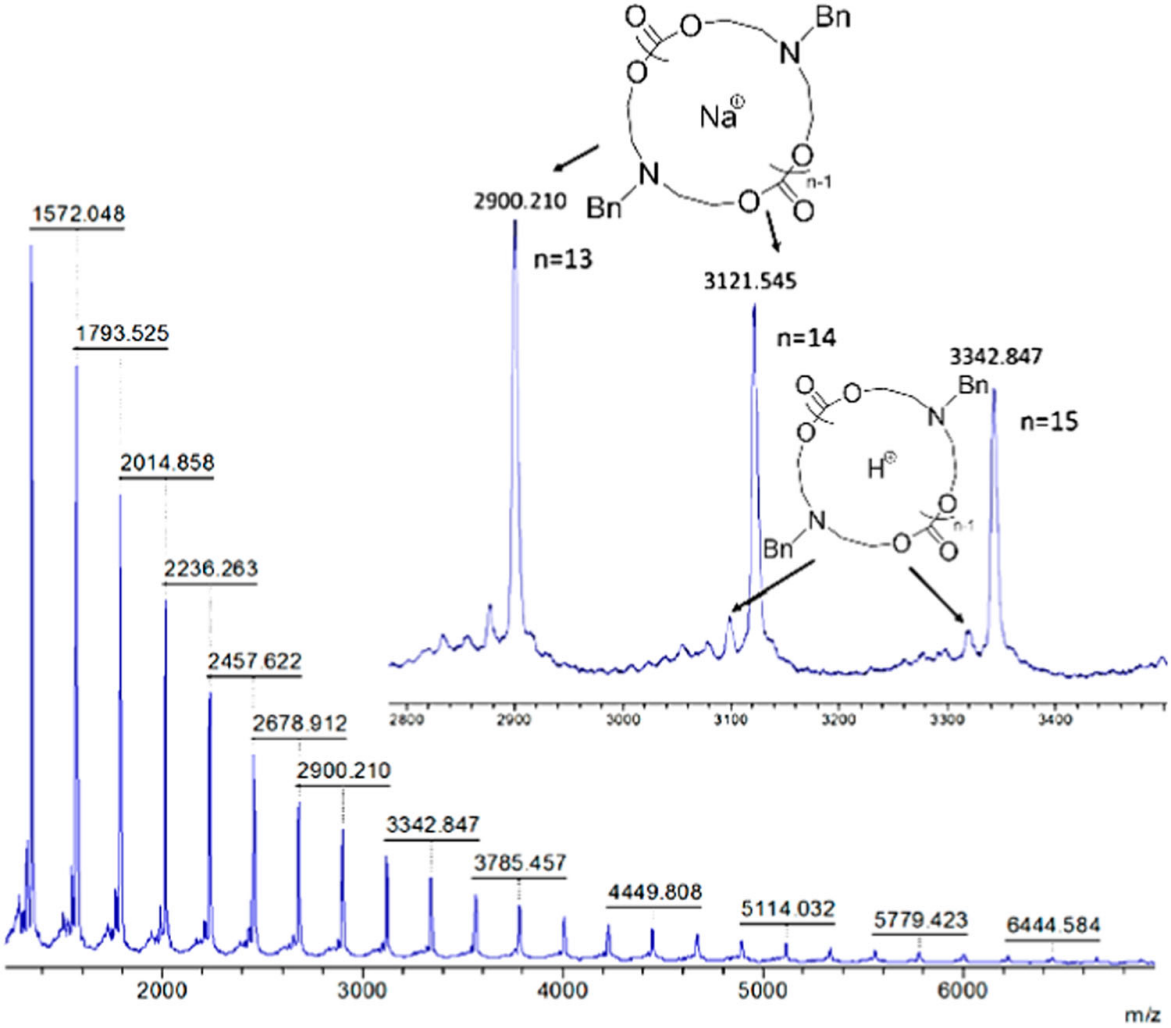
Matrix‐assisted laser desorption/ionization‐mass spectrometry spectrum of *N*‐heterocyclic carbenes mediated 8CC_Bn_. Adapted from Chang et al. (2016) with ACS's permission. [Color figure can be viewed at wileyonlinelibrary.com]

Chang et al. (2016) synthesized cyclic polycarbonates via ZREP. For this project, they studied four *N*‐functionalized eight‐membered cyclic carbonates (8CC) as monomer in THF while using different NHCs as the catalyst at room temperature. But only *N*‐benzyl‐substituted and *N*‐phenyl‐substituted eight‐membered cyclic carbonates (8CC_Bn_ and 8CC_Ph_) show cyclization with NHC‐3 (Scheme 4) as catalysts because ZREP depends on the structures of both NHC and the substituent on the 8CC nitrogen (Scheme 19). The cyclic structures were confirmed by intrinsic viscosity measurement and MALDI‐MS spectra. In the case of 8CC_Bn_, the MALDI‐MS spectrum shows the products were almost entirely cyclic with Na^+^ and H^+^ as the cations (Figure 18), which is also supported by the intrinsic viscosity measurements. They only provided low MWs samples MALDI‐MS spectrum where the cyclic product was detectable between 1.2 and 6.5 kDa (Figure 18). On the other hand, 8CC_Ph_ exhibits crystalline dimerization because the conformational limitation of the planar *N*‐aryl nitrogen prefers dimerization instead of polymerization. To confirm this, the authors conducted a copolymerization reaction between 8CC_Ph_ and δ‐VL. The generation of the cyclic copolymer from this reaction shows that the combination of 8CC_Ph_ and δ‐VL is not favorable for dimerization. MALDI‐MS and intrinsic viscosity measurements confirmed the cyclic copolymer formation. In MALDI‐MS spectrum for the phenyl substituent, there are two noticeable repeating units, 207.23 Da (8CC_Ph_) and 100.12 Da (δ‐VL), which confirm the copolymer. Likewise, the 8CC_Bn_‐VL copolymer was synthesized and evident by MALDI‐MS and intrinsic viscosity measurements. In MALDI‐MS spectrum for the benzyl substituent, there are also two noticeable repeating units, 221.3 Da (8CC_Bn_) and 100.12 Da (VL). Furthermore, the % conversion versus time study for both copolymers also show different results. In the case of 8CC_Ph_‐VL copolymer, 8CC_Ph_ polymerized faster than δ‐VL, but in 8CC_Bn_‐VL copolymer, δ‐VL polymerized much faster than 8CC_Bn_, implying the formation gradient copolymer.

**Figure 19:**
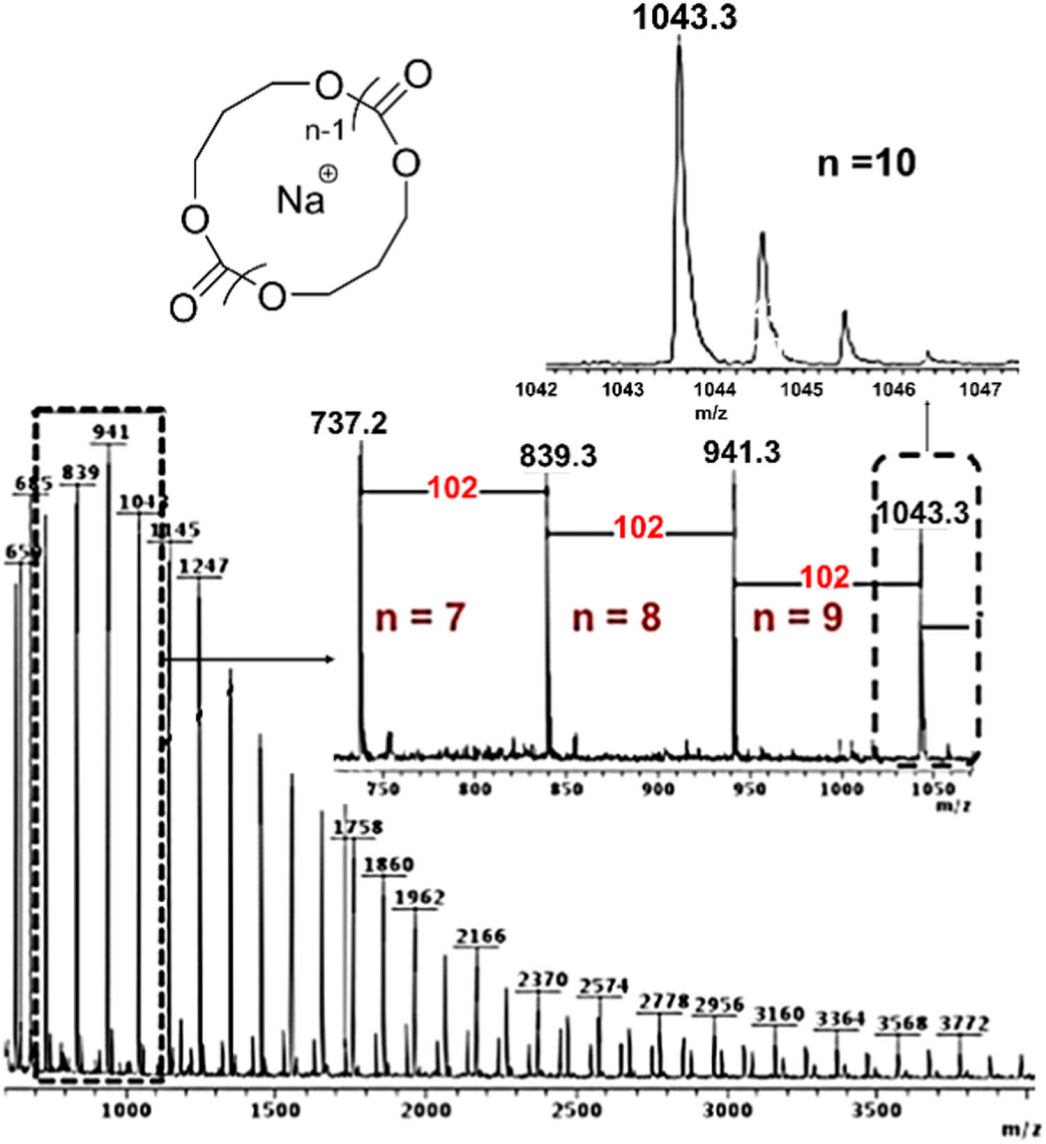
Matrix‐assisted laser desorption/ionization‐mass spectrometry spectrum cyclic poly(TMC) initiated by 1,5,7‐triazabicyclo‐[4.4.0]‐dec‐5‐ene. Adapted from Azemar et al. (2021) with MDPI's permission. [Color figure can be viewed at wileyonlinelibrary.com]

Azemar et al. (2021) achieved cyclic polycarbonates by polymerizing trimethylene carbonate (TMC) catalyzed by 1,5,7‐triazabicyclo‐[4.4.0]‐dec‐5‐ene (TBD) (Scheme 4) in THF via ZREP. The cyclic structure was confirmed by MALDI‐MS, SEC, and ^1^H NMR. The cyclic products were found on the MALDI‐MS spectrum up to 4000 Da (Figure 19).

### 5.2 ZREP of cyclic polysiloxanes

**Scheme 20:**
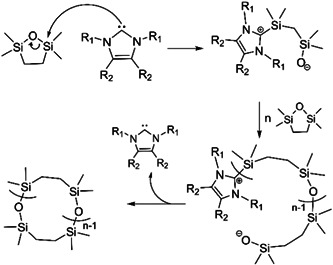
Zwitterionic ring‐expansion polymerization of 2,2,5,5‐tetramethyl‐2,5‐disila‐1‐oxacyclopentane with *N*‐heterocyclic carbenes catalyst. Adapted from Brown et al. (2013) with ACS's permission.

**Figure 20:**
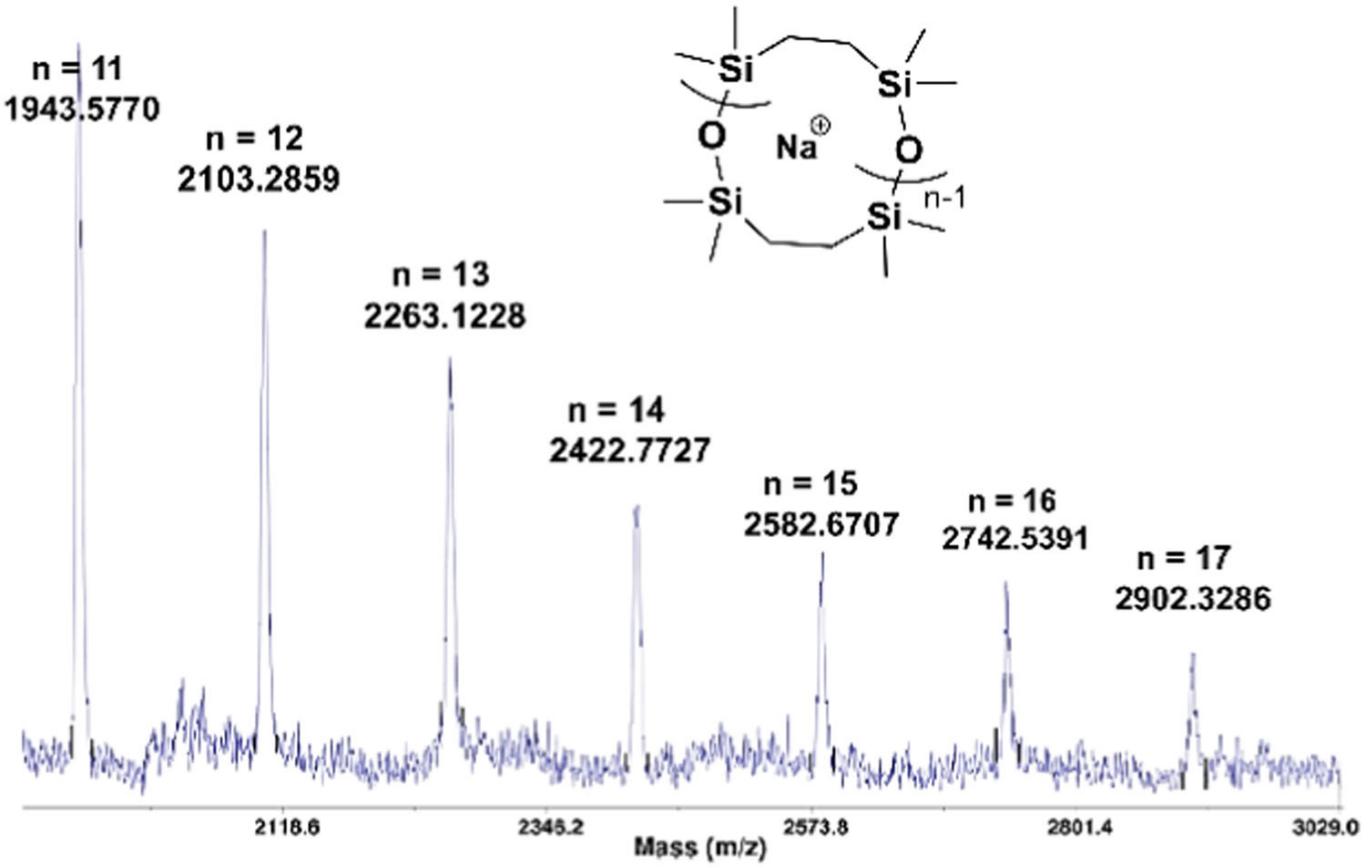
Matrix‐assisted laser desorption/ionization‐mass spectrometry spectrum of *N*‐heterocyclic carbenes mediated poly(TMOSC). Adapted from Brown et al. (2013) with ACS's permission. [Color figure can be viewed at wileyonlinelibrary.com]

In 2013 Brown et al. (2013) synthesized cyclic polysiloxane via ZREP from 2,2,5,5‐tetramethyl‐2,5‐disila‐1‐oxacyclopentane (TMOSC) using NHC‐1, NHC‐3, NHC‐4 (Scheme 4) as catalysts at room temperature. Polymerization rates were much faster with NHC‐1 (5 s) and NHC‐3 (5 s) compared to NHC‐4 (5–30 min) due to their high nucleophilicity. They proposed a ZREP mechanism of the carbene attacking silicone to create zwitterionic intermediates, which eventually propagated to form cyclic polysiloxanes (Scheme 20). Cyclic structures were evident via intrinsic viscosity measurement and MALDI‐MS spectra. The intrinsic viscosity ratio between cyclic and linear analogs was 0.67–0.70, which indicates the product was largely cyclic. MALDI‐MS spectrum of lower MW polysiloxanes generated by catalyzing with NHC‐3 for 24 h, confirmed the formation of cyclic product with number average MW (M_*n*_) of 3100 Da (Figure 20). Depending on the reaction times and monomer‐to‐catalyst ratios: NHC‐4, NHC‐3, and NHC‐1 (Scheme 4) mediated reactions generate cyclic polysiloxanes with MWs (27–86 kDa), 587, and 942 kDa respectively, by SEC.

**Scheme 21:**
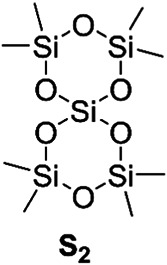
Compound **S**_**2**_. Adapted from Wu et al. (2017) with RSC's permission.

As a second addition to cyclic polysiloxanes Wu et al. (2017) synthesized these via ZREP from compound **S**_**2**_ (Scheme 21) while using TBD (Scheme 4) as the catalyst. The purpose behind synthesizing compound **S**_**2**_ was to increase the ring strain to make it more feasible for ZREP. The cyclic structure was confirmed by NMR, atomic force microscope (AFM), scanning electron microscope (SEM), and DFT calculations. However, no mass spectroscopic analyses were provided. According to SEC, cyclic polysiloxanes with MWs 32–1039 kDa were achieved depending on the reaction times and monomer‐to‐catalyst ratios.

### 5.3 ZREP of cyclic poly(alkylene phosphates)

**Scheme 22:**
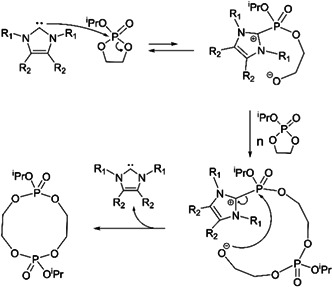
Zwitterionic ring‐expansion polymerization of cyclic poly(iPP) with *N*‐heterocyclic carbenes catalyst. Adapted from Stukenbroeker et al. (2014) with ACS's permission.

**Figure 21:**
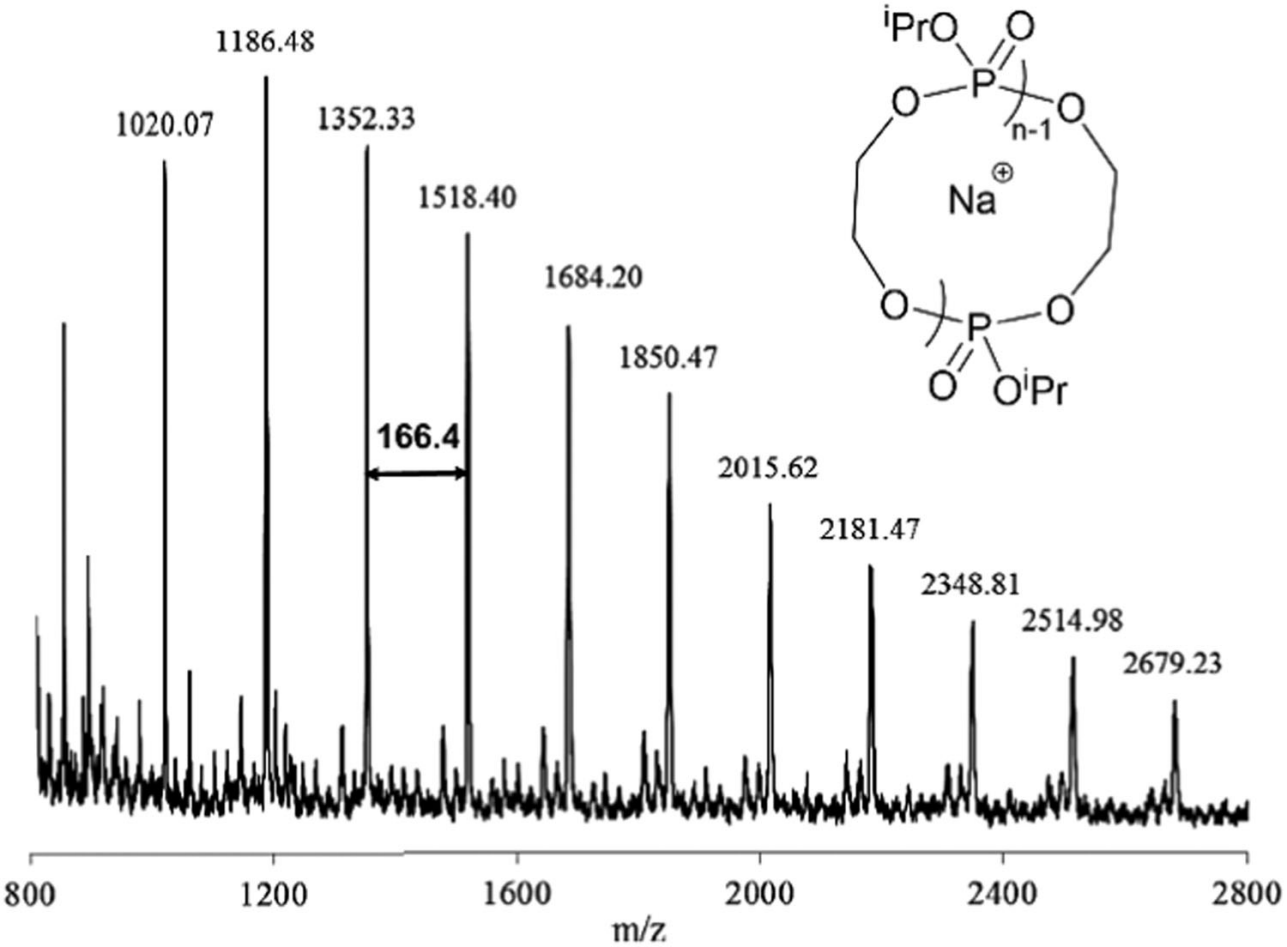
Matrix‐assisted laser desorption/ionization‐mass spectrometry spectrum of NHC‐3 mediated cyclic poly(iPP). Adapted from Stukenbroeker et al. (2014) with ACS's permission.

The first cyclic poly(alkylene phosphates) synthesis was reported by Stukenbroeker et al. (2014) using ZREP. In this project, 2‐isopropoxy‐2‐oxo‐1,3,2‐dioxaphospholane (iPP) was polymerized in THF and toluene in the presence of carbenes (NHC‐1 and NHC‐3) (Scheme 4) as catalysts (Scheme 22). Lower MWs cyclic poly(alkylene phosphates) mediated by NHC‐3 were identified by the MALDI‐MS spectrum (Figure 21) between 800 and 2800 Da. The lower intensity peaks in MALDI‐MS were attributed to the exchange of the isopropyl group with hydrogen since no end group signal was detected on ^1^H and ^31^P NMR. According to the light scattering measurements, they obtained cyclic product up to 202 kDa. The authors could not conduct intrinsic viscosity measurements to confirm the cyclic structure of higher MW due to the failure of generating high MWs linear analogs. However, based on previous “topological entrapment” studies on the cross‐linked gel by Semlyen and coworkers (Fyvie et al., 1987; Wood et al., 1993), they left the polymer in the gel for 11 days, and 36% of the higher MWs polymer was trapped in the gel, indicating a substantial amount of cyclic formation.

## 6 CONCLUSION

ZREP is one of the more advantageous approaches to polymerizing numerous cyclic polymers, including polyesters, polyamides, and polyethers. In this approach, a suitable catalyst can initiate the polymerization to form a zwitterionic intermediate, which can eventually be neutralized to yield the cyclic polymer by eliminating the catalyst during the termination step. One can comprehend the reaction mechanism with detailed examinations of mass spectrometric data, specifically MALDI‐MS. In the case of ZREP of cyclic polyesters, the reaction mechanism and purity of the products primarily depend on the nature of the catalyst. NHCs mediated syntheses for polyesters were the most effective for generating cyclic polymers compared to the other catalysts (such as DBU, ITU, DABCO, NHOs, and Sn complexes). NHCs are also an excellent catalyst for ZREP of cyclic polyamides, although finding the appropriate monomer and the initiator is still challenging. Other catalysts like DBU and pyridine have also been examined for cyclic polyamides, but the product was mostly impure (a mixture of linear & cyclic products). To synthesize cyclic polyether, tris(pentafluorophenyl)borane [B(C_6_F_5_)_3_] is the only catalyst to this day to be used. However, in this case, one has to account for the formation of the tadpole, a portion of which has the exact same MW as the cyclic polyether. More recently, combinations of UPLC, MALDI‐MS/MS fragmentation, and IMS‐MS have been utilized to determine the ratio of “tadpole” versus cyclic architectures. In addition, a “click” scavenging process can remove the unwanted impurities by functionalizing these with a “clickable” group and make sure the product is largely cyclic. Other side reactions, including numerous linear and other cyclic products can be examined by IMS‐MS to determine the extent to which these are extended or contracted. Although this borane catalyst works marginally well, other catalysts must be explored for more pure cyclic polyether. In conclusion, ZREP is an effective method for cyclic polymerization; however, future work can be directed toward other different types of monomers, such as sulfoxide compounds, while searching for more effective catalysts.

## AUTHOR CONTRIBUTIONS

**Mahi Ahmad**: Writing—original draft; writing—review and editing. **Scott M. Grayson**: Project administration; supervision; writing—review and editing.
